# Intramolecular charge transfer enables highly-efficient X-ray luminescence in cluster scintillators

**DOI:** 10.1038/s41467-023-38546-x

**Published:** 2023-05-22

**Authors:** Nan Zhang, Lei Qu, Shuheng Dai, Guohua Xie, Chunmiao Han, Jing Zhang, Ran Huo, Huan Hu, Qiushui Chen, Wei Huang, Hui Xu

**Affiliations:** 1 MOE Key Laboratory of Functional Inorganic Material Chemistry, School of Chemistry and Material Science, Heilongjiang University, 74 Xuefu Road, Harbin, 150080 China; 2 MOE Key Laboratory for Analytical Science of Food Safety and Biology, State Key Laboratory of Photocatalysis on Energy and Environment, College of Chemistry, Fuzhou University, Fuzhou, China; 3 Hubei Collaborative Innovation Centre for Advanced Organic Chemical Materials, Hubei Key Lab on Organic and Polymeric Optoelectronic Materials, Department of Chemistry, Wuhan University, 299 Bayi Road, Wuhan, 430072 P. R. China; 4 Frontiers Science Center for Flexible Electronics (FSCFE), Shaanxi Institute of Flexible Electronics (SIFE), Northwestern Polytechnical University (NPU), 127 West Youyi Road, Xi’an, 710072 China

**Keywords:** Optical materials, Organic LEDs, Applied optics

## Abstract

Luminescence clusters composed of organic ligands and metals have gained significant interests as scintillators owing to their great potential in high X-ray absorption, customizable radioluminescence, and solution processability at low temperatures. However, X-ray luminescence efficiency in clusters is primarily governed by the competition between radiative states from organic ligands and nonradiative cluster-centered charge transfer. Here we report that a class of Cu_4_I_4_ cubes exhibit highly emissive radioluminescence in response to X-ray irradiation through functionalizing biphosphine ligands with acridine. Mechanistic studies show that these clusters can efficiently absorb radiation ionization to generate electron-hole pairs and transfer them to ligands during thermalization for efficient radioluminescence through precise control over intramolecular charge transfer. Our experimental results indicate that copper/iodine-to-ligand and intraligand charge transfer states are predominant in radiative processes. We demonstrate that photoluminescence and electroluminescence quantum efficiencies of the clusters reach 95% and 25.6%, with the assistance of external triplet-to-singlet conversion by a thermally activated delayed fluorescence matrix. We further show the utility of the Cu_4_I_4_ scintillators in achieving a lowest X-ray detection limit of 77 nGy s^−1^ and a high X-ray imaging resolution of 12 line pairs per millimeter. Our study offers insights into universal luminescent mechanism and ligand engineering of cluster scintillators.

## Abstract (web summary)

X-ray luminescence efficiency of copper halide clusters is limited by nonradiative charge transfer. Here, Zhang et al. report highly emissive radioluminescence of Cu4I4 cubes by functionalizing biphosphine ligands with acridine, unlocking the potential use as low-cost and eco-friendly scintillators.

## Introduction

Solution-processed scintillators have recently received significant attention for their ability to transform X-rays into visible light, making them promising candidates for the development of next-generation, high-performance X-ray detectors in medical imaging and industrial detection^1–4^. In particular, metal clusters stabilized by organic ligands possess inherent advantages in achieving effective radioluminescence, given that heavy-atom metal elements in the cluster exhibit superior X-ray absorption properties^5–7^, and their associated organic ligands typically exhibit high rates of radiative transition^8–11^. However, these ligand-capped clusters exhibit both molecular and atomic/ionic electronic characteristics^12–15^, resulting in complex and volatile emissive behaviors^16–21^. For instance, copper clusters, specifically Cu_4_I_4_ cubes^22–24^, are effective in luminescent applications due to their highly rigid cubic structures that suppress John-Teller distortion of excited Cu^+^ ions and reduce structural relaxation-induced energy loss^25–29^. Unfortunately, many copper clusters exhibit changeable emissions dependent on stimuli such as pressure^30^, friction^22^, and temperature variation^31^, since they possess multiple charge-transfer excited states, including metal/iodine-to-ligand (M/ILCT) and intramolecular charge-transfer (LCT) states, as well as triplet metal-to-iodine (^3^MICT) charge-transfer states, also known as cluster-centered (^3^CC) states^32^. It is noteworthy that the presence of low-energy ^3^MICT states can induce multi-channel non-radiative decays, which can significantly impact the luminescent performance of copper clusters^33–36^.

**Fig. 1:**
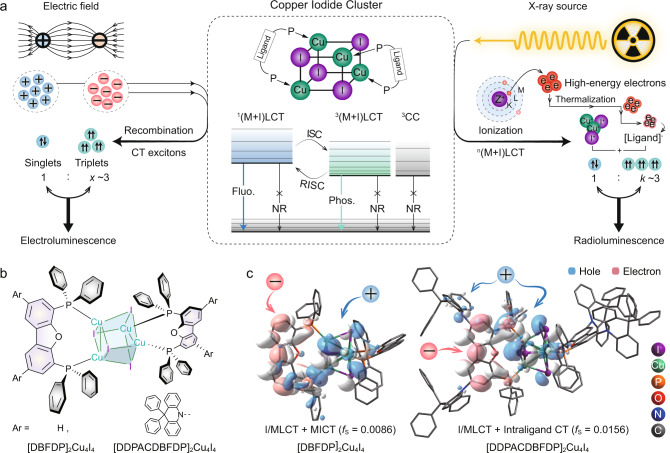
Photo-, electro- and radio-luminescence from Cu_4_I_4_ cubic scintillators. **a** Emission mechanisms of Cu_4_I_4_ cubic clusters. Middle: excited-state composition of Cu_4_I_4_ cubic clusters stabilized with biphosphine ligands. (M + I)LCT and CC refer to metal and iodide-to-ligand charge transfer and cluster centered metal-to-iodide charge transfer (MICT) excited states, respectively, which induce separated positive and negative charges respectively on Cu_4_I_4_ cube and peripheral ligands. Superscripts of “1” and “3” denote singlet and triplet states, respectively. Fluorescence, phosphorescence, nonradiation, intersystem crossing and reverse intersystem crossing are abbreviated as Fluo., Phos., NR, ISC and RISC, respectively. Left: in sandwiched light-emitting diode, hole and electron generated by electric field can be captured by Cu_4_I_4_ cube and ligands to form charge transfer (CT) excitons, corresponding to (M + I)LCT excited states of the clusters, which are then de-excited for electroluminescence (EL). Right: through photoelectric effect and Compton scattering, the inner electrons of Cu and I as heavy atoms are firstly excited by X ray to eject high-energy electrons and ionize Cu_4_I_4_ cube. The hot electrons further interact with atoms to produce massive secondary electrons. After losing sufficient energy, electrons are finally captured by ligands to form hole-electron pairs. The excited clusters characteristic of (M + I)LCT emit radioluminescence (RL). *x* and *k* are ratios of triplet and singlet excitons, and ionized and directly excited molecules, respectively. For both electric field and X-ray excitations, molecular ionization namely (M + I)LCT is dominant, leading to *x* and *k* approaching to three according to spin statistics. **b** Chemical structures of biphosphine stabilized Cu_4_I_4_ cubes, respectively named [DBFDP]_2_Cu_4_I_4_ (Ar = H) and [DDPACDBFDP]_2_Cu_4_I_4_ (Ar = 9,9-diphenylacridine, DPAC). **c** Single crystal structures of the clusters, whose cubes are highlighted in ball-stick model, and contours of triplet “hole” (blue) and “electron” (pink) for nature transition orbitals (NTO) simulated with time-dependent density functional theory (TDDFT). *f*_S_ is singlet oscillator strength.

In a typical cluster scintillator, X-rays ionize Cu_4_I_4_ to release high-energy electrons that initiate secondary electron cascades through atomic interaction^1,37^. These hot electrons quickly become thermalized and are captured by the organic ligands in the cluster scintillator, producing hole-electron pairs that are predominantly characteristic of M/ILCT and LCT states. Under the case of ultraviolet excitation, Cu_4_I_4_ cubes exhibit photoluminescence through a process in which larger distinction coefficient organic ligands are primarily excited to generate M/ILCT and LCT states, followed by energy transfer to low-lying MICT states^9,38^. The situation of electroluminescence from cluster light-emitting diodes (CLED) is similar to radioluminescence in that ligand-involved charge transfer excited states should still predominate^39,40^. Despite electrons mainly being captured by ligands, Cu_4_I_4_ with lower ionization potential can directly capture holes, which may increase nonradiative ^3^MICT under electric excitation. Hence, we reason that precise control over the intramolecular charge-transfer in Cu_4_I_4_ clusters is likely to facilitate efficient radioluminescence through electron and hole transfer from Cu_4_I_4_ to ligands while reducing direct excitation and energy transfer to MICT quenching states (Fig. 1a)^1,9,37,38^. Effective ligand contributions to both “electrons” and “holes” are required at the first singlet (S_1_) and triplet (T_1_) states, along with designing coordination rigidity to achieve high molecular rigidity of ligands, minimize structural relaxation-induced energy loss, and enhance X-ray irradiation stability^40–42^.

## Results

To validate our hypothesis, we designed [DDPACDBFDP]_2_Cu_4_I_4_ (DDPACDBFDP = 10,10'-(4,6-bis(diphenylphosphanyl) dibenzofuran-2,8-diyl)bis(9,9-diphenylacridine)), a cluster with ligands have two rigid, strongly electron-donating 9,9-diphenylacridine (DPAC) groups (Fig. 1b). Due to the DPAC groups being perpendicular to the dibenzofuran moiety, the lone electron pair of the nitrogen atom in the former is not conjugated with the latter. DPAC donors have little influence on the coordination characteristics of the DBFDP moiety. Single-crystal X-ray diffraction confirmed that [DDPACDBFDP]_2_Cu_4_I_4_ preserved a cubic core with nearly unchanged Cu…I and Cu…Cu bond lengths (Fig. 1c and Supplementary Fig. 1). In this sense, DPAC donor would enhance ligand-centered charge transfer, but has little influence on X-ray absorption by MICT. To figure out how DPAC donors influence the electronic structure of the cluster, we simulated the ground-state (S_0_) and transition characteristics of [DBFDP]_2_Cu_4_I_4_ and [DDPACDBFDP]_2_Cu_4_I_4_ with density functional theory (DFT) (Fig. 1c and Supplementary Fig. 2). At S_0_ states, both two clusters show similar highest occupied (HOMO) and lowest unoccupied molecular orbitals (LUMO) located on Cu_4_I_4_ and dibenzofuran, respectively. Introducing DPAC enhances LCT, therefore deepening the LUMO energy level by 0.33 eV. Consistent with this, the experimentally measured HOMO and LUMO energy levels of [DDPACDBFDP]_2_Cu_4_I_4_ are simultaneously deepened by ~0.3 eV, reflecting the significant contributions of its peripheral ligands to charge injection/transfer (Supplementary Fig. 3 and Table S1). For the singlet and triplet excitations, “electrons” are centralized on dibenzofuran groups of the clusters. “Holes” of [DBFDP]_2_Cu_4_I_4_ are localized on phosphine atoms and Cu_4_I_4_. In contrast, “holes” of [DDPACDBFDP]_2_Cu_4_I_4_ partially disperse to DPAC donors. With increased singlet M/ILCT and LCT components, oscillator strength (*f*_S_) of the S_1_ state for [DDPACDBFDP]_2_Cu_4_I_4_ is nearly doubled. This rationally mixed S_1_ and T_1_ states make [DDPACDBFDP]_2_Cu_4_I_4_ able to integrate the advantages of MICT in X-ray absorption and M/ILCT and LCT in emission.

**Fig. 2:**
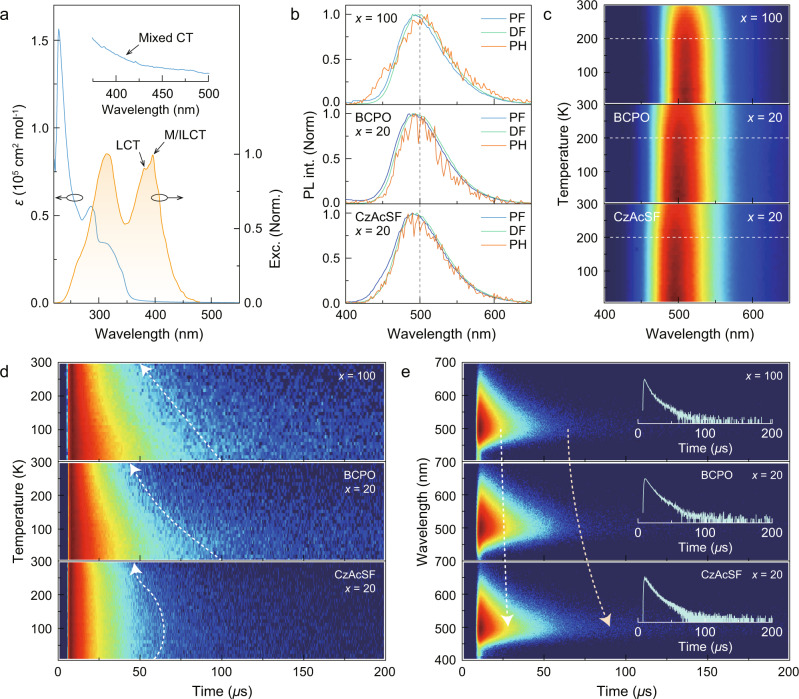
Optical investigations of [DDPACDBFDP]_2_Cu_4_I_4_-based spin-coated films. **a** Electronic absorption (blue line) and excitation (yellow line) spectra of neat [DDPACDBFDP]_2_Cu_4_I_4_ film. Absorption tail in 400–500 nm is magnified, originated from M/ILCT. **b** Prompt (PF) and delayed fluorescence (DF) and phosphorescence (PH) spectra of host:*x*% [DDPACDBFDP]_2_Cu_4_I_4_ films recorded in time ranges of <1 µs, 1–200 µs and >200 µs, respectively (*x* = 100 for neat film, and 20 for doped films). BCPO is bis-4-((N-carbazolyl)phenyl)phenylphosphine oxide as a high-triplet-energy host, and CzAcSF is 10-(4-((4-(9H-carbazol-9-yl)phenyl)sulfonyl)-phenyl)−9,9-dimethyl-9,10-dihydroacridine as a thermally activated delayed fluorescence (TADF) featured host. **c** Temperature dependence of steady-state emission spectra for host:*x*% [DDPACDBFDP]_2_Cu_4_I_4_ films. **d** Temperature dependence of time decays for delayed fluorescence (DF) from host:*x*% [DDPACDBFDP]_2_Cu_4_I_4_ films. Dash arrows mark the variation tendencies along with temperature increasing. **e** Time-resolved transi**e**nt emission (TRES) spectra of host:*x*% [DDPACDBFDP]_2_Cu_4_I_4_ films at room temperature. Light blue and yellow dash arrows respectively show the differences regarding to prompt fluorescence (PF) and DF contributions. Insets are time decay curves at peak.

The electronic absorption spectra of [DDPACDBFDP]_2_Cu_4_I_4_ film exhibited mixed charge transfer absorption tails in the range of 350-500 nm (inset of Fig. 2a), indicating the presence of ligand-to-metal charge transfer (LMCT) and metal-to-ligand charge transfer (MLCT) transitions. Excitation spectra of the two clusters revealed the dominant contribution of ligand-centered charge transfer states to singlet and triplet radiative transitions (Fig. 2a and Supplementary Figs. 4–5). For [DBFDP]_2_Cu_4_I_4_, its MICT excitation spectrum also included a M/ILCT-attributed band caused by increased ligand-to-Cu_4_I_4_ energy transfer. Luminescence of both clusters depended on their respective ligand-centered charge transfer transitions. When doped in traditional BCPO and thermally activated delayed fluorescence (TADF) CzAcSF matrices at a concentration of 20%, the photoluminescence peaks of [DDPACDBFDP]_2_Cu_4_I_4_ slightly shift blue by 5–10 nm compared to its neat film (Supplementary Fig. 6). However, prompt fluorescence (PF), delayed fluorescence (DF), and phosphorescence (PH) spectra of [DDPACDBFDP]_2_Cu_4_I_4_-based films are comparable at room temperature, indicating near-zero singlet-triplet splitting for effective reverse intersystem crossing (Fig. 2b). Furthermore, [DDPACDBFDP]_2_Cu_4_I_4_ exhibited temperature-independent photoluminescence profiles, indicating its stable excited-state composition (Fig. 2c and Supplementary Figs. 7–8). In contrast, [DBFDP]_2_Cu_4_I_4_ neat film showed that with temperature increasing, its ligand-involved emission bands at ~450 nm slightly enhanced, while the emission intensity of its MICT band at ~550 nm increased and then decreased, indicating a turning point at 230 K due to worsened triplet quenching at high temperature^43^ (Supplementary Fig. 7).

The BCPO matrix effectively suppresses intermolecular interaction-induced quenching (Supplementary Fig. 8b), leading to an increases in the photoluminescence quantum yield (PLQY, *ϕ*_PL_) from 71% for the neat cluster film to 85% for BCPO:[DDPACDBFDP]_2_Cu_4_I_4_. Further improvement of the PLQY is achieved with the CzAcSF matrix, which provides additional reverse intersystem crossing for triplet-to-singlet conversion, thus reducing triplet concentration quenching and achieving a state-of-the-art *ϕ*_PL_ of 95% for CzAcSF:[DDPACDBFDP]_2_Cu_4_I_4_. Consistent with this, the decay lifetimes of the doped films (τ_1_ = ~ 5.5 µs; τ_2_ = ~ 15.0 µs) at room temperature are longer than the neat films (τ_1_ = ~ 3.5 µs; τ_2_ = ~ 12.0 µs) (Fig. 2d and Supplementary Figs. 9–13). Furthermore, the CzAcSF-hosted film reveals that decay lifetimes first increase from 20 K to 150 K and then slightly decrease at 150-300 K (Supplementary Fig. 10). This is attributed to the complementarily contributed triplet utilization of CzAcSF and the M/ILCT and LCT states of the cluster. In this case, the triplet capture by MICT states of clusters is further limited.

Time-resolved emission spectra (TRES) indicate that the BCPO matrix effectively prevents influences of intermolecular interactions on photoluminescence properties of [DDPACDBFDP]_2_Cu_4_I_4_, extending the time range of high photon density (in red and orange colors) to 25 µs (Fig. 2e). Using the CzAcSF matrix slightly increases time range of high photon density to 30 µs, due to reverse intersystem crossing and Förster energy transfer^44^. In contrast to the MICT-originated PH component of [DBFDP]_2_Cu_4_I_4_ neat film, which is directly proportional to temperature (Supplementary Fig. 14), TRES profiles of [DDPACDBFDP]_2_Cu_4_I_4_-based films are independent on temperature, reflecting limited MICT components (Supplementary Fig. 15). Moreover, CzAcSF matrix stabilizes TRES intensities and contours of the clusters, in contrast to the variability observed with neat and BCPO-hosted films. The predominance of M/ILCT and LCT components is crucial for achieving high luminescence stability and quantum efficiency, which is also in accord to the requirement of X-ray excited emission on highly radiative CT excitons. Therefore, compared to [DBFDP]_2_Cu_4_I_4_, [DDPACDBFDP]_2_Cu_4_I_4_ with more efficient M/ILCT and LCT excited components can be superior for radioluminescence applications.

**Fig. 3:**
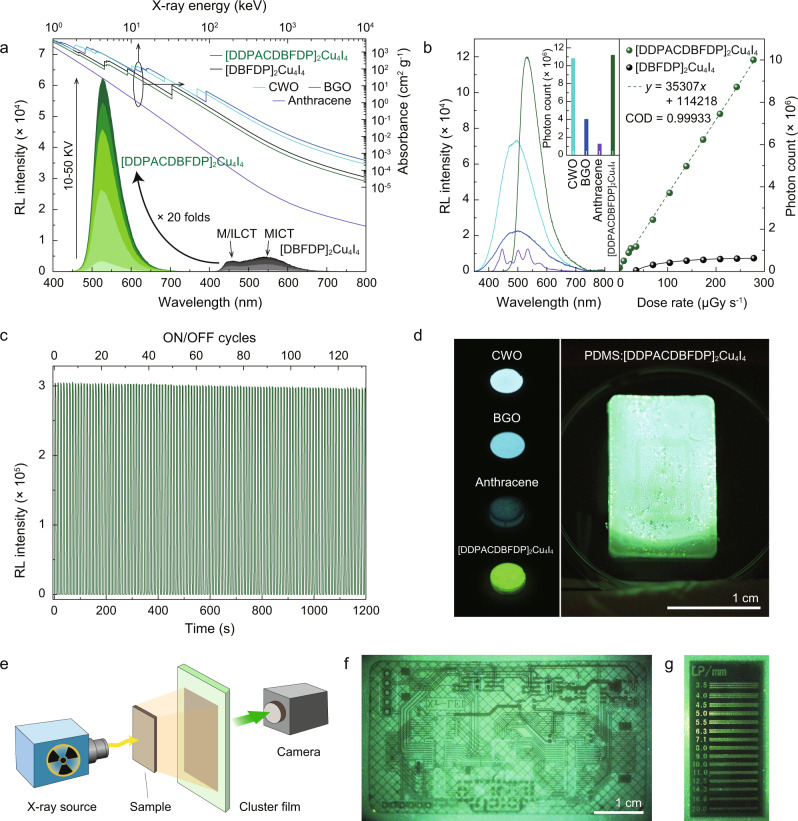
Radioluminescence properties of the Cu_4_I_4_ cubic scintillators. **a** X-ray absorption spectra of [DDPACDBFDP]_2_Cu_4_I_4_, [DBFDP]_2_Cu_4_I_4_ and commercial scintillators (cadmium tungstate (CWO), bismuth germinate (BGO) and anthracene) and dependence of radioluminescence spectra for clusters on X-ray tube voltage. **b** radioluminescence spectra and corresponding total radioluminescence photon numbers of [DDPACDBFDP]_2_Cu_4_I_4_ and the commercial scintillators at a dose rate of 278 µGy_air_ s^−1^, and comparison on total radioluminescence photon number variation of the clusters at different dose rates. The radioluminescence photon number-dose rate relationship of [DDPACDBFDP]_2_Cu_4_I_4_ is linearly fitted. COD refers to coefficient of determination. Detection limit is estimated with 3σ/slope method^47^. **c** radioluminescence stability of [DDPACDBFDP]_2_Cu_4_I_4_ under on-off cycles of X-ray radiation at a dose rate of 278 µGy_air_ s^−1^. **d** Photographs of pure scintillator samples (left), and polydimethylsiloxane (PDMS) hosted [DDPACDBFDP]_2_Cu_4_I_4_ film (right) excited by X-ray radiation (X-ray tube voltage, 50 kV; dose rate, 104 µGy_air_ s^−1^). **e** Schematic of the electronic X-ray imaging by cluster scintillator films. A large-scale PDMS:[DDPACDBFDP]_2_Cu_4_I_4_ film serves as the detector placed between a X-ray source and a digital camera. **f** X-ray image of an integrated circuit board recorded at 50 kV. **g** High-resolution X-ray image of a gauge for line-pairs per millimeter (LP/mm) enabled by [DDPACDBFDP]_2_Cu_4_I_4_ (voltage, 50 kV).

To investigate the radioluminescence properties of the clusters, we measured X-ray absorption and emission spectra (Fig. 3). The X-ray absorption coefficients of Cu_4_I_4_ and P atoms (*Z* = 53 and *K*_α_ = 33.2 keV for iodine; *Z* = 29 and *K*_α_ = 9.0 keV for copper; *Z* = 15 and *K*_α_ = 2.142 keV for phosphorous) are far greater than those of C, H, N and O atoms (*Z* = 1–8, *K*_α_ = 0.0136–0.531 keV). Furthermore, the high absorbance of the copper-iodine clusters indicates the predominance of their coordination skeletons in X-ray absorption. This is evidenced by not only the identical resonant absorption edges of [DDPACDBFDP]_2_Cu_4_I_4_ and [DBFDP]_2_Cu_4_I_4_ but also their X-ray absorbance being significantly larger than that of commercial organic scintillator Anthracene (Fig. 3a). Moreover, we found that the X-ray absorbability of the clusters is directly correlated to the proportions of their coordination skeletons. As a result, the absorption coefficient of [DDPACDBFDP]_2_Cu_4_I_4_, which has larger ligands, is markedly lower than that of [DBFDP]_2_Cu_4_I_4_ in X-ray energy range of 1-10^4^ keV. However, in the contrast to their X-ray absorbance, we found that the radioluminescence intensity of [DDPACDBFDP]_2_Cu_4_I_4_ is 20-fold greater than that of [DBFDP]_2_Cu_4_I_4_. This indicates that the photon conversion efficiency of [DDPACDBFDP]_2_Cu_4_I_4_ is significantly higher than that of [DBFDP]_2_Cu_4_I_4_. We also observed that the peak radioluminescence intensity of [DDPACDBFDP]_2_Cu_4_I_4_ is even stronger than that of commercial inorganic scintillators cadmium tungstate (CWO) and bismuth germinate (BGO), despite the latter having 1-2 orders of magnitude larger X-ray absorbance (Fig. 3b). Finally, we calculated that the radioluminescence photon number of [DDPACDBFDP]_2_Cu_4_I_4_ is 1.04, 1.77, and 8.69 times greater than those of CWO, BGO, and anthracene, respectively, highlighting its potential to practical applications.

The radioluminescence photon number of [DBFDP]_2_Cu_4_I_4_ remained nearly unchanged when the X-ray tube voltage exceeded 30 kV, which is attributed to concentration quenching of its MICT state (Fig. 3a). In contrast, [DDPACDBFDP]_2_Cu_4_I_4_ exhibited continuously increased radioluminescence photons, achieving a 7-fold increase at 50 kV. Importantly, in the range of 0.688–278 µGy S^−1^, the radioluminescence photon number of [DDPACDBFDP]_2_Cu_4_I_4_ is was indirectly proportional to dose rate (Fig. 3b and Supplementary Figs. 16−17). Conversely, the increase of radioluminescence photon number for [DDPACDBFDP]_2_Cu_4_I_4_ was markedly reduced at dose rates exceeding 139 µGy S^−1^, indicating worsened concentration quenching. Consequently, at 278 µGy S^−1^, [DDPACDBFDP]_2_Cu_4_I_4_ ehibited a luminescence signal ~16 times stronger than [DBFDP]_2_Cu_4_I_4_. Thus, enhanced luminescence from ligand-centered charge transfer states proved to be the key factor inducing largely improved scintillator characteristics of [DDPACDBFDP]_2_Cu_4_I_4_. This results also imlies that exciton formation and allocation follow the charge recombination mechanism after X-ray excitation. Furthermore, the high-intensity X-ray-excited luminescence and linear dependence of photon number on X-ray dose rate led to an low X-ray detection limit of 77 nGy S^−1^, which is <2% of the standard X-ray diagnostic dosage of 5.5 µGy S^−1,^^45^. Even after exposure under 278 µGy S^−1^ for 20 min, the radioluminescence intensity of [DDPACDBFDP]_2_Cu_4_I_4_ remained stable, verifying the high X-ray photostability of cubic Cu_4_I_4_ clusters (Fig. 3c). Additionally, [DDPACDBFDP]_2_Cu_4_I_4_ demonstrated good photo- and thermo- stabilities and water tolerance, making it competent for diverse applications in complicated and harsh conditions (Supplementary Fig. 18).

We further demonstrates the utility of [DDPACDBFDP]_2_Cu_4_I_4_ for flexible X-ray radiography by incorporating the cluster scintillators into a polydimethylsiloxane (PDMS) film (Fig. 3d). This cluster-doped PDMS film was then utilized as a detector placed between an integrated circuit board and a digital camera (Fig. 3e). Under X-ray excitation, the details of integrated circuit can be visualized (Fig. 3f). The high resolution of X-ray image was further verified using line pairs per millimeter gauge tests (Fig. 3g). Even at 12 line pairs per millimeter, the line gap can still be clearly recognized clearly, corresponding to a resolution of <40 μm.

**Fig. 4:**
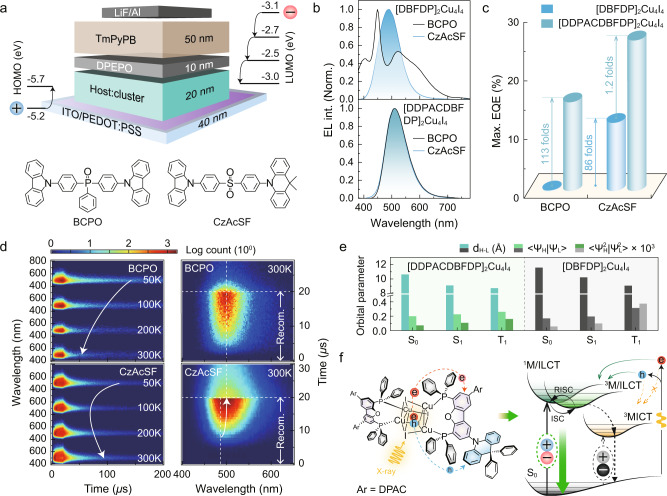
Hole-electron-pair coupling for luminescence from Cu_4_I_4_ clusters. **a** Device configuration and energy level structure of spin-coated cluster light-emitting diodes (CLED) and chemical structures of the hosts BCPO and CzAcSF. HOMO and LUMO refer to the highest occupied and the lowest unoccupied molecular orbitals. **b** Electroluminescence spectra of CLEDs respectively using BCPO (top) and CzAcSF (bottom) as hosts. **c** Comparison on the maximum external quantum efficiencies of the CLEDs. **d** Electroluminescence TRES contours (left) of [DDPACDBFDP]_2_Cu_4_I_4_ based devices at 50, 100, 200 and 300 K, and emission contours at recombination stage (≤ 20 µs) after applying a 5 V bias pulse at room temperature (right). Arrows show the variation tendencies of DF components. **e** Comparison on centroid distance (d_H_-_L_), wave function overlap integral (<Ψ_H_ | Ψ_L_ >) and electronic cloud overlap probabilities (<Ψ_H_^2^ | Ψ_L_^2^ >) of the LUMOs and the HOMOs for ground states (S_0_) and the “hole” and “electron” for the S_1_ and T_1_ states of the clusters. **f** Proposed mechanism of enhanced radioluminescence for [DDPACDBFDP]_2_Cu_4_I_4_, in comparison to [DBFDP]_2_Cu_4_I_4_.

To gain further understanding of the impact of ligand-involved charge transfer transitions on charge recombination process, the electroluminescence properties of the clusters were investigated. Owing to the similarity of radioluminescence and electroluminescence mechanisms with respect to hole-electron pair utilization, trilayer CLEDs were fabricated through spin-coating clusters in BCPO and CzAcSF matrices, respectively (Fig. 4a, b and Supplementary Figs. 19–21). The CzAcSF matrix rendered a 50-fold increase in *η*_EQE_ for [DBFDP]_2_Cu_4_I_4_, reaching 11.6%, owing to limited MICT. In addition to a state-of-the-art *η*_EQE_ of 25.6% in CzAcSF matrix, the maximum *η*_EQE_ of [DDPACDBFDP]_2_Cu_4_I_4_ in BCPO matrix (15.1%) was 75-fold of that of [DBFDP]_2_Cu_4_I_4_ (0.2%), verifying that the predominance of ligand-centered charge transfer states for the former largely improved exciton radiation (Fig. 4c and Supplementary Figs. 22–24). It is noteworthy that the maximum internal quantum efficiency (IQE, *η*_IQE_) of CzAcSF:[DDPACDBFDP]_2_Cu_4_I_4_ reached 100%, which was consistent with its *ϕ*_PL_. On the contrary, BCPO:[DDPACDBFDP]_2_Cu_4_I_4_ had the maximum *η*_IQE_ of ~60%, which was only three quarters of its *ϕ*_PL_. This manifests that triplet quenching in BCPO-based devices was more serious than in the corresponding films, due to a larger initial triplet ratio in the devices (75%). Therefore, for radioluminescence process with the same triplet/singlet ratio, the predominance of ligand-centered charge transfer states for [DDPACDBFDP]_2_Cu_4_I_4_ gives rise to its markedly improved radioluminescence performance.

We further performed exciton kinetic analysis to further understand the roles of charge transfer transitions on hole-electron pairs (Fig. 4d). Electroluminescence TRES contours show that electroluminescence lifetimes of BCPO:[DDPACDBFDP]_2_Cu_4_I_4_ based devices were shortened at higher temperature (Fig. 4d, left and Supplementary Fig. 24), while electroluminescence lifetime variation of CzAcSF:[DDPACDBFDP]_2_Cu_4_I_4_ has a turning point at 100 K (Supplementary Fig. 25). The increased electroluminescence decays at ≥100 K originate from TADF characteristics of CzAcSF, indicating the crucial contribution of CzAcSF matrix to triplet-to-singlet conversion and exciton utilization. We further compared the stages of carrier recombination, energy transfer, and radiation during electroluminescence processes at room temperature (Fig. 4d, right). Transient emissions from BCPO:[DDPACDBFDP]_2_Cu_4_I_4_ based devices corresponded to pure emissions of the cluster, which were time-independent, and started at ~2 µs. On the contrary, in carrier recombination stage, electroluminescence radiation from CzAcSF:[DDPACDBFDP]_2_Cu_4_I_4_ was postponed by ~3 µs, which was taken for reverse intersystem crossing by CzAcSF (reverse intersystem crossing rate constant of 8.5 × 10^4^ s^−1^)^46^. Furthermore, sky-blue emission from CzAcSF was observed at the beginning. It means excitons were firstly formed on CzAcSF. Then, electroluminescence emission gradually shifted to cluster-originated bluish green (508 nm) at the end of recombination stage and whole decay stage, demonstrating the key advantage of CzAcSF in converging excitons to ligand-centered charge transfer states.

Our study shows that the significant increase in the radioluminescence intensity of [DDPACDBFDP]_2_Cu_4_I_4_ is coherent with its significantly larger *ϕ*_PL_ and *η*_EQE_. Additionally, MICT-attributed bands in the radioluminescence spectra of [DBFDP]_2_Cu_4_I_4_ are considerably stronger than the photoluminescence spectra but comparable to electroluminescence spectra, which implies a similarity between the radioluminescence and electroluminescence mechanisms in exciton formation through charge recombination. To further understand the correlation between the charge transfer characteristics and radioluminescence performance, we compared frontier molecular orbital (FMO) properties of the clusters (Fig. 4e and Supplementary Fig. 26). The larger FMO overlaps of the S_0_ and S_1_ states for [DDPACDBFDP]_2_Cu_4_I_4_ favor radiative transition, resulting in stronger radioluminescence emission. Simultaneously, triplet “hole” of [DDPACDBFDP]_2_Cu_4_I_4_ is widely distributed on DPAC and partial P…Cu-I moieties apart from the benzofuran-centralized triplet “electrons”. This triplet electronic structure provides an efficient charge-releasing channel from Cu_4_I_4_ cube to locally coordinated skeleton during thermalization and generates ligand-involved charge transfer excitons (Fig. 4f). On the other hand, MICT-predominant triplet state of [DBFDP]_2_Cu_4_I_4_ induces the majority of excitons always trapped by its Cu_4_I_4_ core. Therefore, high radioluminescence performance of [DDPACDBFDP]_2_Cu_4_I_4_ is mainly due to the dominant population of its highly radiative M/ILCT and LCT states.

## Discussion

Our study has developed a general ligand-engineering strategy to significantly enhance X-ray luminescent performance of Cu_4_I_4_ cubic scintillators through the construction of ligand-centered charge-transfer excited states. Our results demonstrate that the predominance of M/ILCT and LCT states over MICT states is crucial for enhancement of radioluminescence in Cu_4_I_4_ clusters. Importantly, the introduction of donor groups in ligands leads to an enhanced emission efficiency by >20-fold for radioluminescence, 14-fold for photoluminescence, and 75-fold for electroluminescence efficiencies in [DDPACDBFDP]_2_Cu_4_I_4_. Moreover, inorganic Cu_4_I_4_ and organic ligands complement each other in the radioluminescence process, where the dominance of Cu_4_I_4_ in X-ray absorption is overshadowed by the effect of donor groups that induce charge release to the ligand-coordinated skeleton. This leads to the predominance of radiative M/ILCT and LCT states in emission that enables for efficient population of ligand-centered charge-transfer states for high luminescence performance. Our findings offer insights for utilizing organic-inorganic hybrid features of clusters for multiple luminescence applications and benefit the design of high-performance cluster scintillators.

## Methods

### Copper iodide cluster synthesis

Clusters were synthesized through dissolving 1 mmol of ligand and 2 mmol of CuI in dichloromethane and stirring for several hours to afford crude powder, which was recrystallized to afford white crystals. Detailed experimental procedures are provided in the Supplementary Information.

### Radioluminescence analysis

An X-ray detector was put on film surface. A beam of X-ray source (P357, VJ Technologies) was applied to the electronic board with different amounts of X-ray exposure. Spectra and images were recorded using a spectrometer and an optical microscope, respectively. Additional measurement details are provided in the Supplementary Information.

### Photoluminescence spectroscopy analysis

Steady-state and transient emission measurements were performed using an Edinburgh FLS 1000 fluorescence spectrophotometer equipped with Xenon lamp for 200–900 nm measurement, nano- and microsecond pulsed lamps for 100 ps-10s measurement and a temperature controller for 11-500 K measurement.

### Electroluminescence analysis

The devices were fabricated through spin-coating for emissive layers and vacuum evaporation for electron-transporting layers and cathodes, respectively. A system composed of a Keithley 4200 source meter, a calibrated silicon photodiode and a PR655 spectrum colorimeter was used to measure voltage-current density-luminance characteristics and electroluminescence spectra. Transient and temperature-dependent electroluminescence spectral measurements were performed with FLS 1000 by incorporating a Tektronix AFG3022G function generator.

## Supplementary information

Supplementary Information

## Data Availability

The authors declare that the data generated in this study are provided in Supplementary Information. The X-ray crystallographic data of [DDPACDBFDP]_2_Cu_4_I_4_ generated in this study have been deposited in the Cambridge Crystallographic Data Centre (CCDC) under deposition number 2201097 [DOI: 10.5517/ccdc.csd.cc2cwf4v].
