# Supplementary material for: Intramolecular charge transfer enables highly-efficient X-ray luminescence in cluster scintillators
Source: Nat Commun. 2023 May 22;14:2901. doi: 10.1038/s41467-023-38546-x (PMC10203249; doi:10.1038/s41467-023-38546-x)
Supplement: Supplementary file 1 — Supplementary Information [file 41467_2023_38546_MOESM1_ESM.pdf]

Supplementary Information

Intramolecular charge transfer enables highly-efficient X-ray luminescence in cluster scintillators

Nan Zhang[1,‡], Lei Qu[1,‡], Shuheng Dai[2,‡], Guohua Xie[3,‡], Chunmiao Han[1], Jing Zhang[1], Ran Huo[1], Huan Hu[1], Qiushui Chen[2,*], Wei Huang[4] & Hui Xu[1,*]

[1]MOE Key Laboratory of Functional Inorganic Material Chemistry & School of Chemistry and Material Science, Heilongjiang University, 74 Xuefu Road, Harbin 150080, China.

[2]Frontiers Science Center for Flexible Electronics (FSCFE) & Shaanxi Institute of Flexible Electronics (SIFE), Northwestern Polytechnical University (NPU), 127 West Youyi Road, Xi'an 710072, China.

[3]Hubei Collaborative Innovation Centre for Advanced Organic Chemical Materials, Hubei Key Lab on Organic and Polymeric Optoelectronic Materials, Department of Chemistry, Wuhan University, 299 Bayi Road, Wuhan 430072, P. R. China.

[4]MOE Key Laboratory for Analytical Science of Food Safety and Biology, State Key Laboratory of Photocatalysis on Energy and Environment, College of Chemistry, Fuzhou University, Fuzhou, China.

[‡]These authors contributed equally

*Correspondence to: qchen@fzu.edu.cn (QC); hxu@hlju.edu.cn (HX).

Table of Contents

I. Materials and Synthesis Method 2

II. Structure Characterization and Physcial Measurements 4

III. Theoretical Simulation 6

IV. Photophysical Analysis 9

V. X-Ray luminescence Analysis 17

VI. Electroluminescence Analysis 22

VII. References 30

I. Materials and Synthesis Method

Reagents and solvents for syntheses were purchased from Aldrich and Alfa-Aesar. All chemicals were used without further purification. $[DBFDP]_2Cu_4I_4$ was prepared according to our previous report[1]. Materials used for device fabrication were purchased from P-OLED technology company, which were used after once more sublimation.

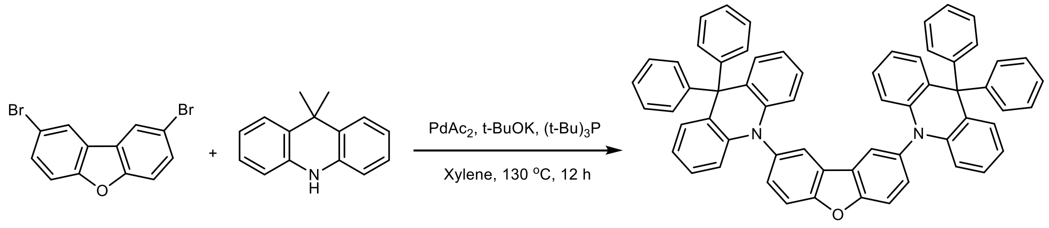

Synthesis of 2,8-bis(9,9-diphenylacridin-10(9H)-yl)dibenzofuran. In Ar, 2,8-Dibromodibenzofuran (16.35 g, 50 mmol), 9,9-diphenyl-9,10-dihydroacridine (36.63 g, 110 mmol), Palladiumacetate (0.625 g, 2.5 mmol), t-BuOK (12.32 g, 110 mmol) and $(t\text{-}Bu)_3P$ (18.75 ml, 7.5 mmol) were mixed in 300 mL of Xylenes and stirred for 12 h at 130 ^{o}C. After the removal of the solvent, the mixture was extracted from water and dichloromethane (3 × 30 mL). The organic layer was combined and dried with anhydrous Na_2SO_4. The solvent was removed in vacuo. The residue was purified by flash column chromatography with the eluant of DCM:PE (1:10) to afford white powder (22.0 g) with a yield of 65%. ^{1}H NMR (TMS, $CDCl_3$, 400 MHz): δ = 7.763-7.742 (d, J = 8.4 Hz, 2H), 7.492-7.487 (d, J = 2 Hz, 2H); 7.287-7.191 (m, 16H); 7.048-7.001 (m, 10H); 6.923-6.858 (m, 8H); 6.434-6.413 (d, J = 8.4 Hz, 4H). LDI-TOF: m/z (%) 830 (100) [M^+]. Elemental Analysis for $C_{62}H_{42}N_2O$: calculated: C 89.61, H 5.09, N 3.37; found: C 89.60, H 5.10, N 3.40.

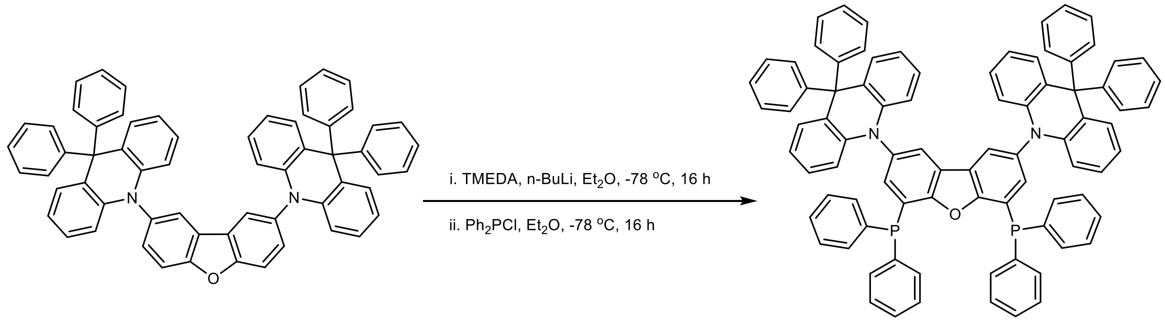

DDPACDBFDP

Synthesis of 10,10'-(4,6-bis(diphenylphosphanyl)dibenzofuran-2,8-diyl)bis(9,9-diphenyl-9,10-dihydroacridine) (DDPACDBFDP). In Ar, 2,8-bis(9,9-diphenylacridin-10(9H)-yl)dibenzofuran (6.14 g, 7.4 mmol) and N,N,N',N'-tetramethylethylenediamine (TMEDA, 5.55 mL, 37 mmol) were dissolved in anhydrous diethyl ether (50 mL) and cooled to -78 ^{o}C. Then, n-butyllithium (2.5 M in hexane, 14.8 mL, 37 mmol) was

added dropwise under stir and further reacted for 16 h. Then, a solution of chlorodiphenylphosphine (7.4 mL, 40.7 mmol) in diethyl ether (10 mL) was added dropwise. The cold bath was removed, and the reaction mixture was stirred for another 16 h. After then, the reaction was quenched with water (50 mL). The mixture was extracted with dichloromethane (3 × 50 mL). The organic layer was combined and dried with anhydrous Na_2SO_4. The solvent was removed in vacuo. The residue was purified by flash column chromatography with the eluant of DCM:PE (1:10) to afford the ligand as white powder of 7.09 g with a yield of 80%. ^{1}H NMR (TMS, $CDCl_3$, 400 MHz): δ = 7.342-7.303 (m, 8H), 7.278-7.237 (m, 14H); 7.216-7.187 (m, 12H); 7.036-6.973 (m, 6H); 6.947-6.927 (m, 8H); 6.856-6.847 (m, 8H); 6.328-6.307 (d, J = 8.4 Hz, 4H). LDI-TOF: m/z (%) 1198 (100) [M^+]. Elemental Analysis for $C_{86}H_{60}N_2OP_2$: calculated: C 86.12, H 5.04, N 2.34; found: C 86.13, H 5.05, N 2.38.

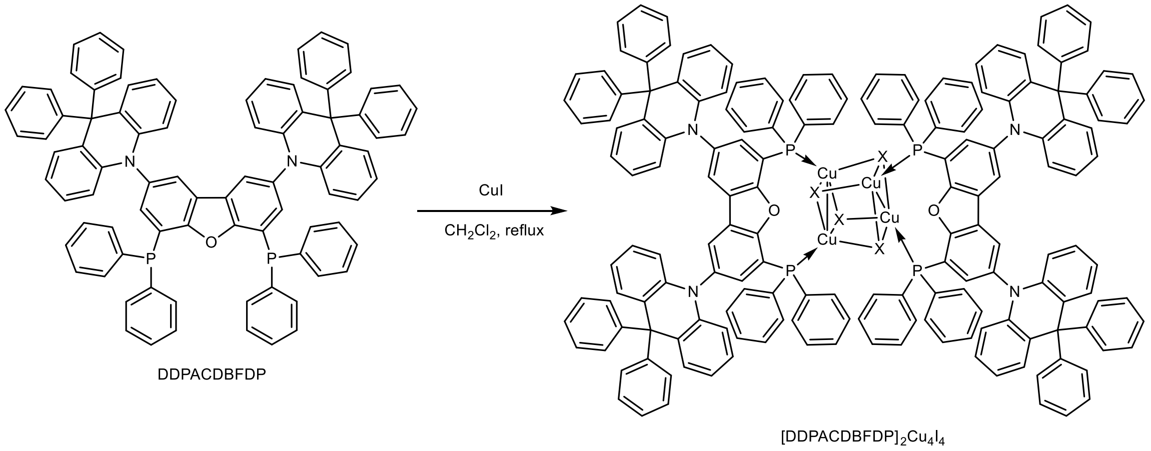

Synthesis of [DDPACDBFDP]$_2$Cu$_4$I$_4$. In Ar, 1 mmol of DDPACDBFDP (1.198 g) and 2 mmol of CuI (380 mg) were dissolved in 20 mL of CH_2Cl_2. The mixture was stirred for 4 h. Then, the solvent was evaporated to obtain crude complex, which was further recrystallization from CH_2Cl_2/ether solution to afford 1.3 g of white crystal with a yield of 82%. ^{1}H NMR (TMS, $CDCl_3$, 400 MHz): δ = 7.655-7.613 (t, J = 8 Hz, 16H), 7.357-7.306 (m, 28H); 7.215-7.182 (m, 26H); 6.936-6.902 (m, 26H); 6.843-6.833 (m, 16H); 6.183-6.163 ppm (d, J = 8 Hz, 8H). LDI-TOF: m/z (%) 3160 (100) [M^+]. Elemental Analysis for $C_{172}H_{120}N_4O_2P_4Cu_4I_4$: calculated: C 65.36, H 3.83, N 1.77, found: C 65.37, H 3.84, N 1.81. CCDC number: 2201097.

II. Structure Characterization and Physcial Measurements

^{1}H NMR spectra were recorded using a Varian Mercury plus 400NB spectrometer relative to tetramethylsilane (TMS) as internal standard. Molecular masses were determined by a FINNIGAN LCQ Electro-Spraying Ionization-Mass Spectrometry (ESI-MS), or a MALDI-TOF-MS. Elemental analyses were performed on a Vario EL III elemental analyzer. The crystal suitable for single-crystal XRD analysis was obtained through slowly diffusing hexane into dichloromethane solution of the clusters at room temperature. All diffraction data were collected at 295 K on a Rigaku Xcalibur E diffractometer with graphite monochromatized Mo Kα (λ = 0.71073 Å) radiation in ω scan mode. All structures were solved by direct method and difference Fourier syntheses. Non-hydrogen atoms were refined by full-matrix least-squares techniques on F2 with anisotropic thermal parameters. The hydrogen atoms attached to carbons were placed in calculated positions with C−H = 0.93 Å and U(H) = 1.2Ueq(C) in the riding model approximation. All calculations were carried out with the SHELXL97 program. Absorption and photoluminescence (PL) emission spectra of the target compound were measured using a SHIMADZU UV-3150 spectrophotometer and a SHIMADZU RF-5301PC spectrophotometer, respectively. Thermogravimetric analysis (TGA) and differential scanning calorimetry (DSC) were performed on Shimadzu DSC-60A and DTG-60A thermal analyzers under nitrogen atmosphere at a heating rate of 10 °C min^{-1}. Cyclic voltammetric (CV) studies were conducted using an Eco Chemie B. V. AUTOLAB potentiostat in a typical three-electrode cell with a glassy carbon working electrode, a platinum wire counter electrode, and a silver/silver chloride (Ag/AgCl) reference electrode.

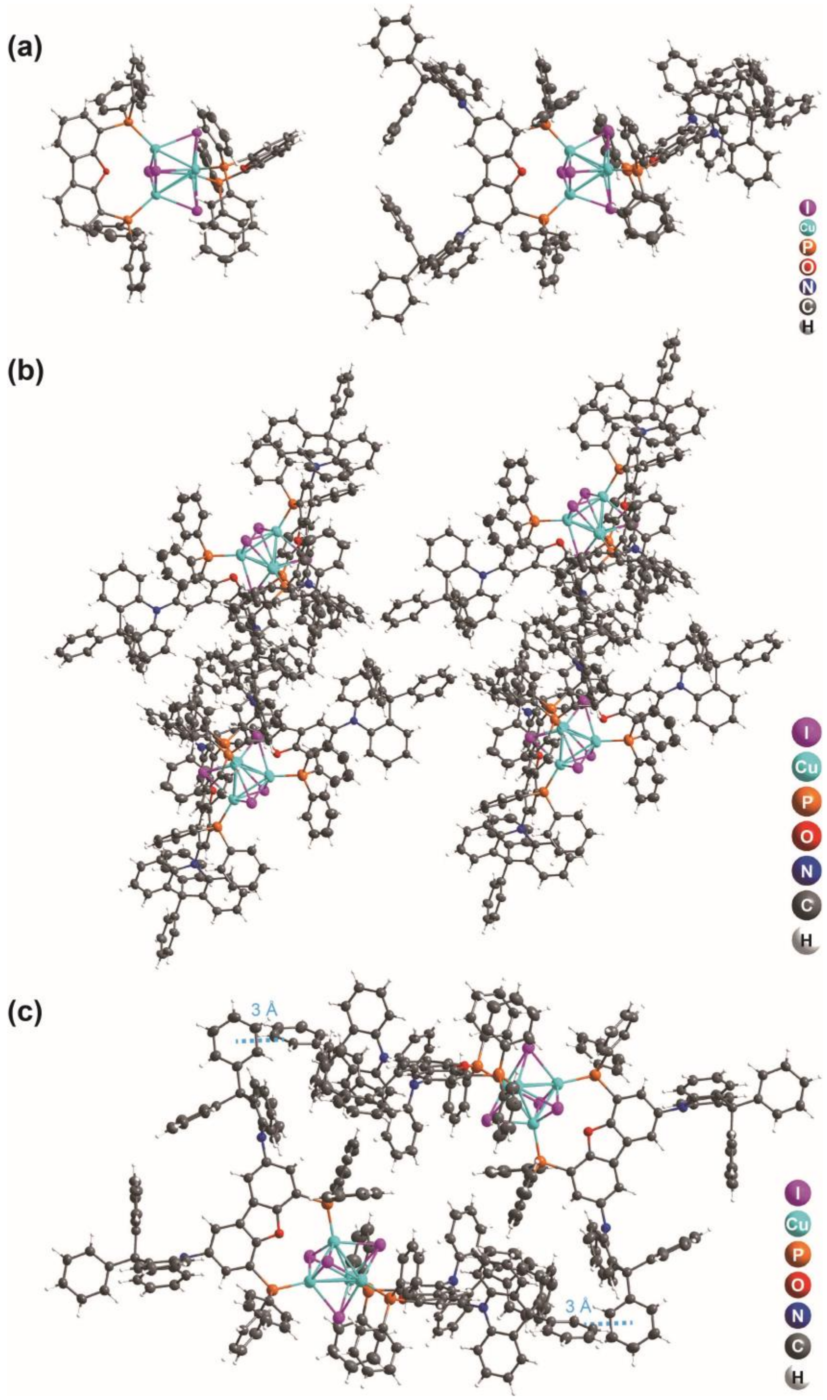

Supplementary Fig. 1 | (a) Single crystal structures of the cluster; and the packing diagrams of (b) $[DBFDP]_2Cu_4I_4$ and (c) $[DDPACDBFDP]_2Cu_4I_4$. It is showed that two clusters have the nearly identical coordination units. DPAC donors hardly change the bond lengths and angles of Cu_4I_4 core. Selected bond lengths (Å) and angles (°): For $[DBFDP]_2Cu_4I_4$: I1-Cu1 2.7047(10), I2-Cu1 2.6861(10), I4-Cu1 2.6725(11), P1-Cu1 2.2379(18), ∠I1-Cu1-I2 108.68(3)^o, ∠I1-Cu1-I4 97.57(3)^o, ∠I2-Cu1-I4 119.87(3)^o, the dihedral angle between two dibenzofuran rings = 72.943(400)^o; For $[DDPACDBFDP]_2Cu_4I_4$: I1-Cu1 2.7129(12), I2-Cu1 2.6529(11), I4-Cu1 2.7042(11), P1-Cu1 2.2413(19), ∠I1-Cu1-I2 102.86(4)^o, ∠I1-Cu1-I4 120.55(4)^o, ∠I2-Cu1-I4 104.79(4)^o, the dihedral angle between two dibenzofuran rings = 78.960(777)^o.

III. Theoretical Simulation

Density functional theory (DFT) and time-dependent DFT (TDDFT) computations were carried out with different parameters for structure optimizations and vibration analyses. The ground state (S_0) configuration was established according to single crystal data. The S_0, singlet and triplet states in vacuum were simulated by the restricted and unrestricted formalism of Beck's three-parameter hybrid exchange functional[2] and Lee, and Yang and Parr correlation functional[3] B3LYP/6-31G(d,p) for ligands and double-ζ LANL2DZ basis sets for CuI, respectively. The fully optimized stationary points were further characterized by harmonic vibrational frequency analysis to ensure that real local minima had been found without imaginary vibrational frequency. The total energies were also corrected by zero-point energy both for the ground state and triplet state. Natural transition orbital (NTO) analysis was performed on the basis of optimized ground-state geometries at the same level.[4] The contours were visualized with Gaussview 5.0. All computations were performed using the Gaussian 09 package.[5]

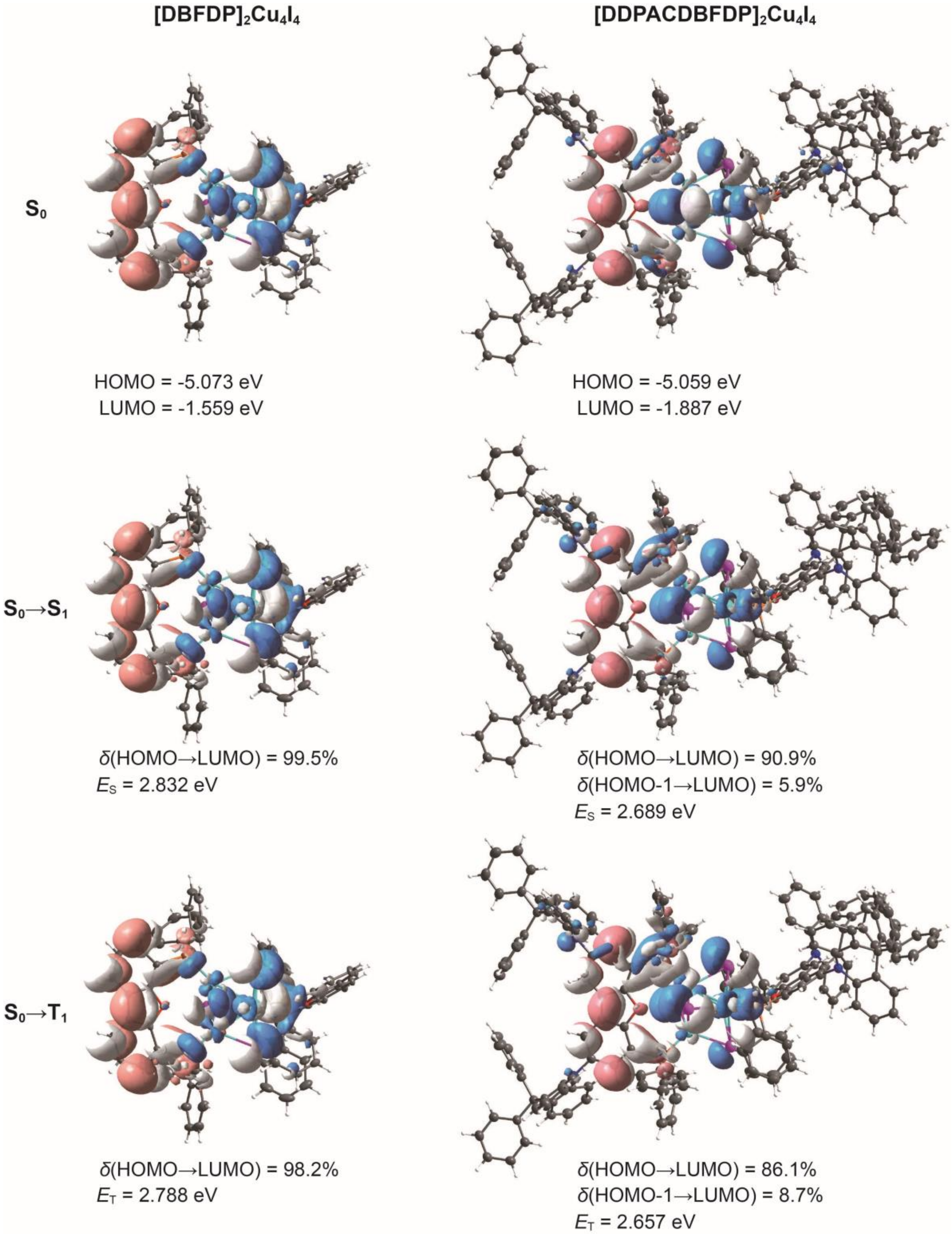

Supplementary Fig. 2 | Orbital and transition parameters and contours of the frontier molecular orbitals of $[DBFDP]_2Cu_4I_4$ and $[DDPACDBFDP]_2Cu_4I_4$ at ground states and singlet/triplet excitations. Pink and blue contour colors refer to the LUMO and the HOMO for the ground (S_0) states and the „electron“ and „hole“ for the singlet and triplet excitations. Compared to $[DBFDP]_2Cu_4I_4$, the highest occupied (HOMO) and lowest unoccupied molecular orbital (LUMO) energy levels of $[DDPACDBFDP]_2Cu_4I_4$ are respectively increased and decreased by 0.014 and 0.328 eV. Therefore, the electron-donating effect of DPAC group not only directly elevates the HOMO energy level of the cluster, but also enhances intramolecular charge transfer interactions,

leading to the reduced LUMO energy level. Different to $[DBFDP]_2Cu_4I_4$, the HOMO of the ground state and „holes“ of the singlet and triplet states for $[DDPACDBFDP]_2Cu_4I_4$ mainly locate on partial phosphine-coordinated Cu_4I_4 core, and partially disperse to DPAC. It is shown that the singlet and triplet excitations of $[DDPACDBFDP]_2Cu_4I_4$ are composed of the HOMO→LUMO transitions with the major weights of 90.9% and 86.1%, and the HOMO-1→LUMO transitions with the minor weights of 5.9% and 8.6%, respectively. The incorporation of the HOMO-1 with significant contribution of DPAC provides the effective hole transfer channel from Cu_4I_4 to ligands during thermalization process. The different contours and DPAC contributions of the singlet and triplet states for $[DDPACDBFDP]_2Cu_4I_4$ enable intersystem crossing for efficient phosphorescence and delayed fluorescence.

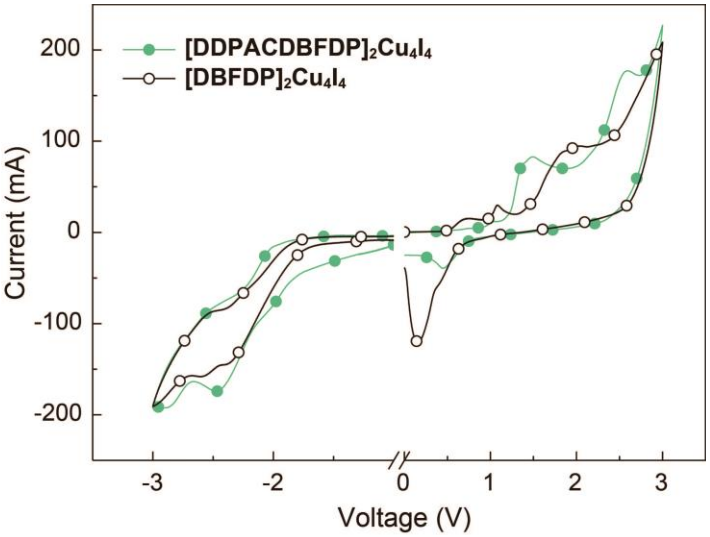

Supplementary Fig. 3 | Cyclic voltammograms of measured at room temperature with the scanning rate of 100 mV s^{-1}. The cyclic voltammetry (CV) analysis of $[DDPACDBFDP]_2Cu_4I_4$ shows that the anodic curve includes two reversible peaks at -2.04 and -2.48 V featuring single-electron reduction of dibenzofuran and DPAC groups, respectively; while, its cathodic curve composes of three irreversible peaks originated from Cu^+, DPAC and dibenzofuran, respectively. It is noted that Cu^+-attributed oxidation peak of $[DDPACDBFDP]_2Cu_4I_4$ is markedly weaker than that of $[DBFDP]_2Cu_4I_4$, since the incorporation of DPAC groups to oxidation. As consequence, according to the onset voltages, the apparent HOMO and LUMO levels of $[DDPACDBFDP]_2Cu_4I_4$ are -5.57 and -3.22 eV, respectively, which are deeper than those of $[DBFDP]_2Cu_4I_4$. Therefore, it is rational that DPAC enhances hole-capture ability and intramolecular charge transfer of ligands, makin DDPACDBFDP more incorporated into carrier injection and exciton formation.

IV. Photophysical Analysis

1. Spectral measurement

Steady-state emission spectra were measured using an Edinburgh FPLS 1000 fluorescence spectrophotometer. Films were prepared by spin coating. Time-resolved emission spectra were measured with Time-Correlated Single Photon Counting (TCSPC) method with a nanosecond hyrogen flash lamp and a microsecond pulsed Xenon light source for 100 ps-10 s lifetime measurement, the synchronization photomultiplier for signal collection and the Multi-Channel Scaling Mode of the PCS900 fast counter PC plug-in card for data processing. A temperature controller was equipped to achieve 11-500 K variation. Spectra of prompt fluorescence (PF), delayed fluorescence (DF) and phosphorescence (PH) were sliced from time-resolved emission spectra (TRES) in the time ranges of <1 μs, 1-100 μs and >150 μs, respectively.

2. Photoluminescence quantum yield measurement

Photoluminescence quantum yields (PLQY, ϕ_{PL}) of these films were measured through a Labsphere 1-M-2 (ϕ = 6") integrating sphere coated with Benflect having efficient light reflection from 200-1600 nm, which was integrated with FPLS 1000. The absolute ϕ_{PL} determination of the sample was performed with two spectral (emission) scans, with the emission monochromator scanning over the Rayleigh scattered light from the sample and a blank substrate. The first spectrum recorded the scattered light and the sample emission, and the second spectrum recorded the scattered light of the Benflect coating. Integration and subtraction of the scattered light in the two spectra are equal to the number of photons absorbed by the samples (N_a), while integration of the sample emission is equal to the number of photons emitted (N_e). Then, absolute ϕ_{PL} can be estimated according to the equation of $\phi_{PL} = N_e/N_a$. Spectral correction (emission arm) was applied to raw data after background subtraction, and from these spectrally corrected curves, the quantum yield was calculated using an F900 software wizard.

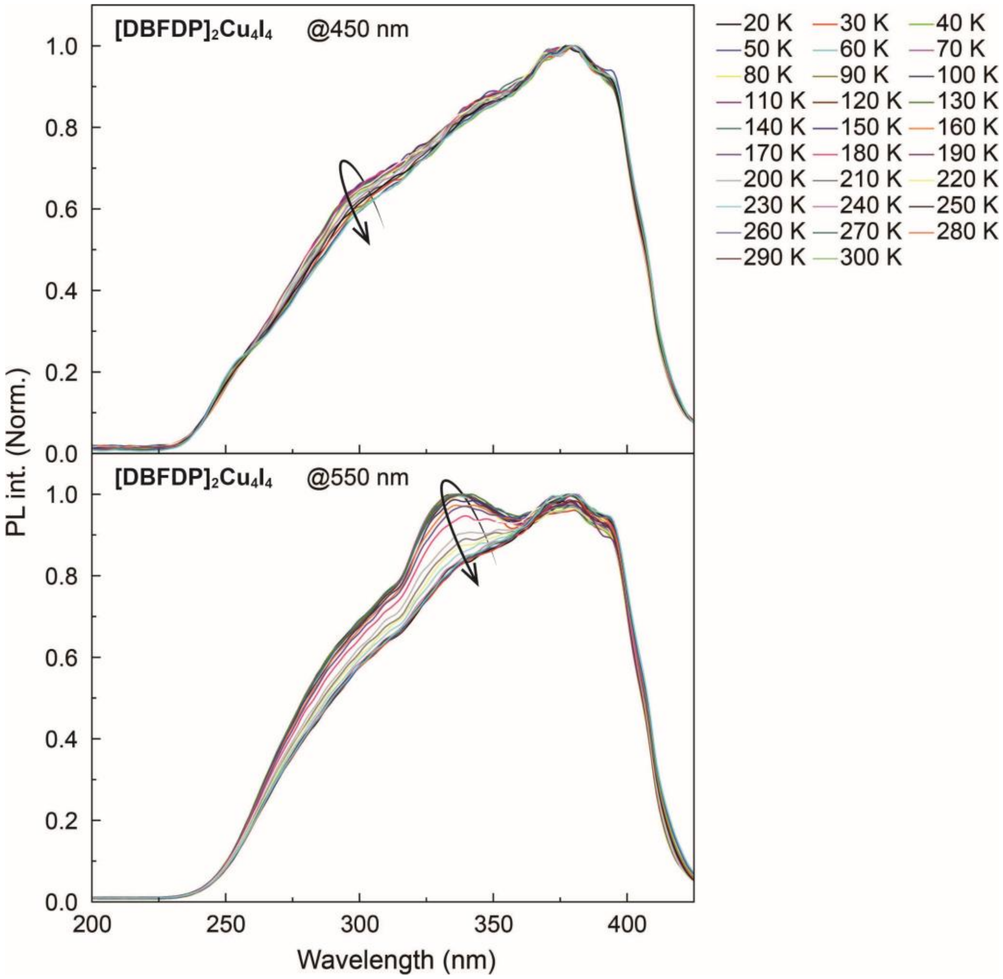

Supplementary Fig. 4 | Temperature-correlated excitation spectra of $[DBFDP]_2Cu_4I_4$ film in the range of 20-300 K.

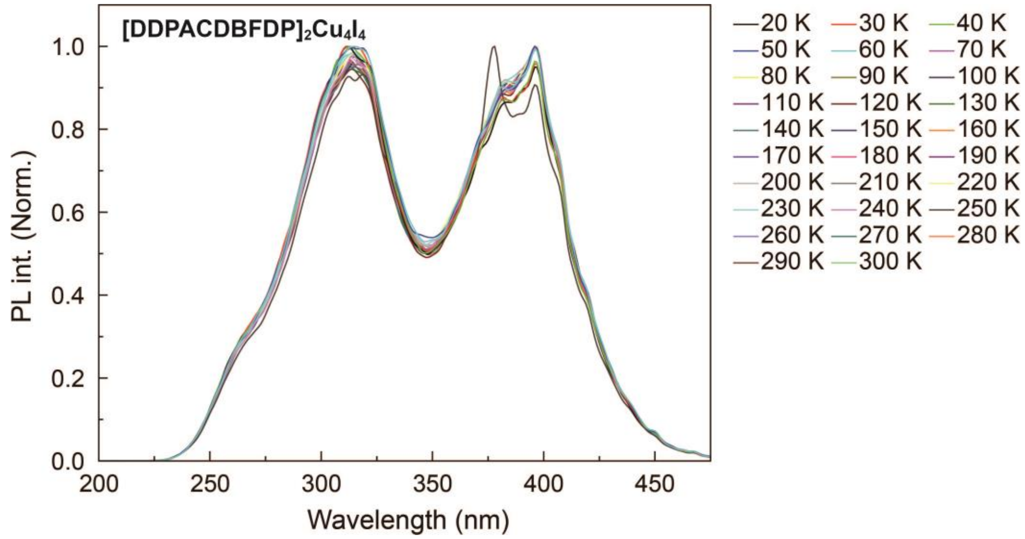

Supplementary Fig. 5 | Temperature-correlated excitation spectra of $[DDPACDBFDP]_2Cu_4I_4$ film in the range of 20-300 K. Different to absorption spectra with weak charge transfer absorption bands, excitation spectra of the clusters mainly contain the LCT and M/ILCT bands at 375-400 nm. $[DDPACDBFDP]_2Cu_4I_4$ shows temperature-independent excitation spectra, owing to suppressed nonradiation at high temperature. Excitation spectra of ligand-centered charge transfer emissions from $[DBFDP]_2Cu_4I_4$ also keep stable, but its ^{3}CC attributed excitation spectra indicate that two cluster-centered bands are weakened at high temperature, due to triplet quenching. At higher temperature, the identical excitation spectra of both ligand- and cluster-centered emissions demonstrate the enhanced energy transfer from high-energy MLCT/ILCT/LCT to MICT and also the superiority of the former in quenching suppression.

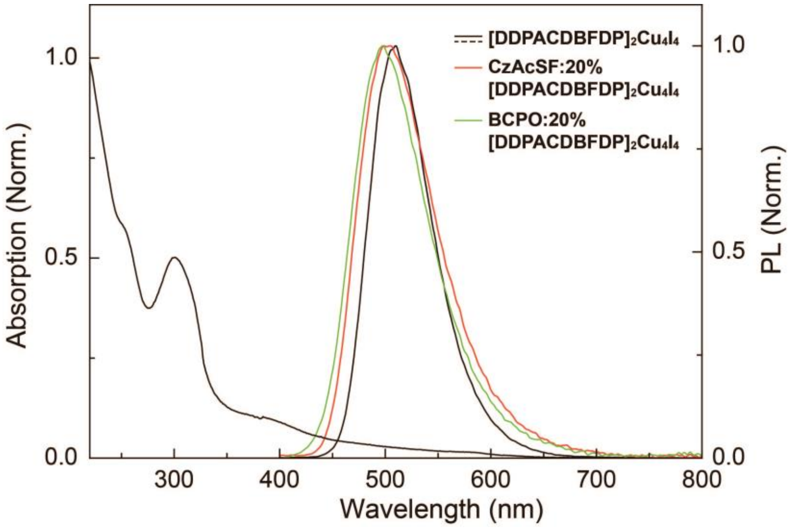

Supplementary Fig. 6 | Electronic absorption spectrum of neat $[DDPACDBFDP]_2Cu_4I_4$ film, and photoluminescence spectra of neat, BCPO and CzAcSF hosted $[DDPACDBFDP]_2Cu_4I_4$ films.In film, the absorption spectrum of $[DDPACDBFDP]_2Cu_4I_4$ preserves the fine structures, owing to steric hindrance of 3D configuration. Nonetheless, Due to stronger molecular packing, photoluminescence peak of neat film is still red shifted, compared to doped films. Furthermore, in contrast to BCPO, CzAcSF with higher molecular polarity also induces a slight emission bathochromic shift.

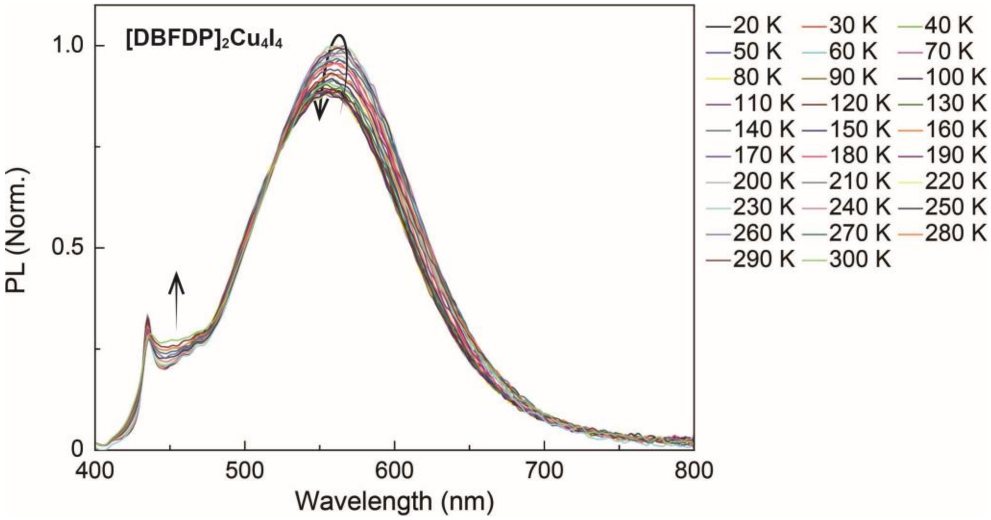

Supplementary Fig. 7 | Temperature dependence of photoluminescence spectra for $[DBFDP]_2Cu_4I_4$ film in the range of 20-300 K.

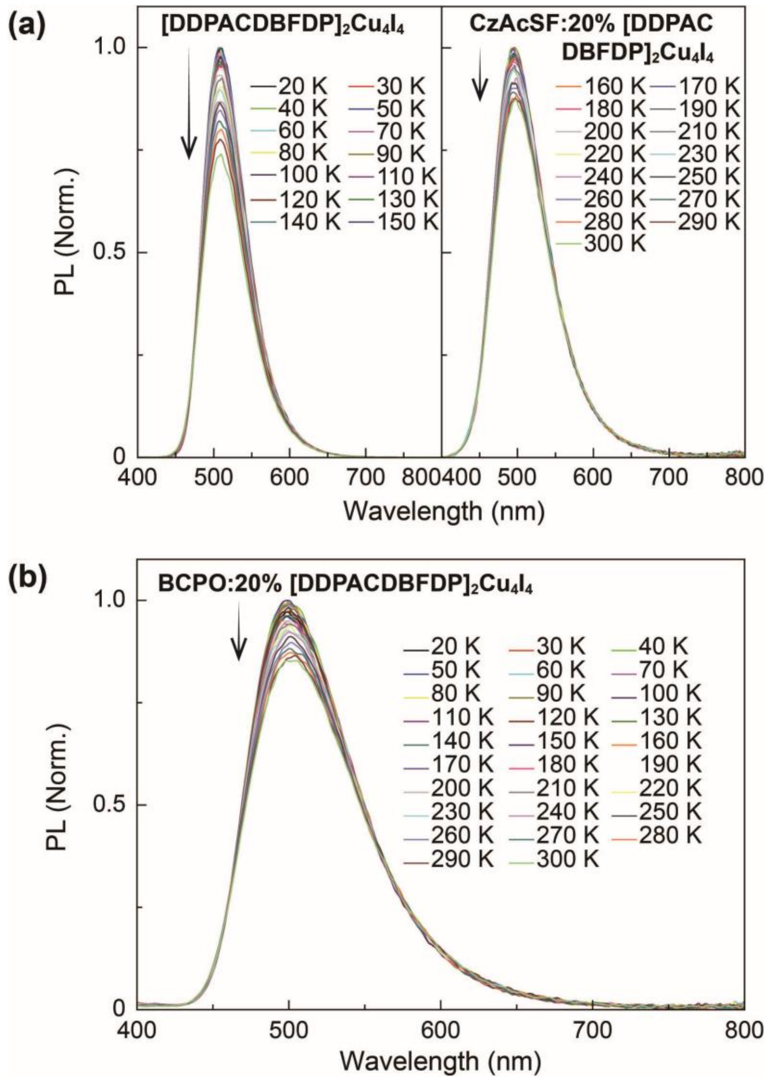

Supplementary Fig. 8 | Temperature dependence of photoluminescence spectra for (a) neat and CzAcSF-hosted $[DDPACDBFDP]_2Cu_4I_4$ films and (b) BCPO-hosted film in the range of 20-300 K.

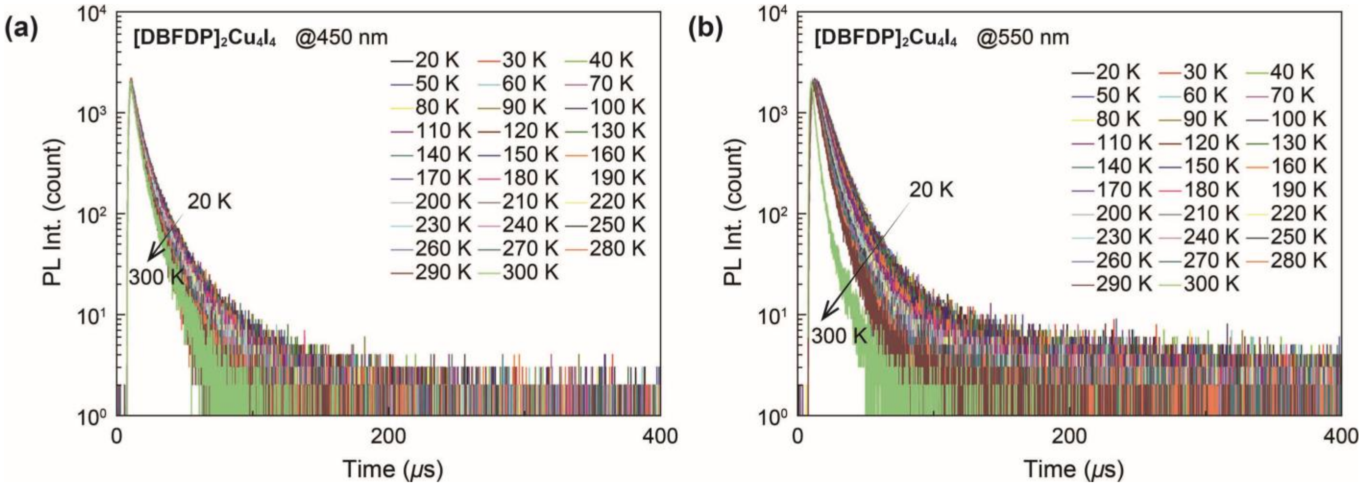

Supplementary Fig. 9 | Temperature dependence of time decays for blue (a) and yellow (b) emission components of neat $[DBFDP]_2Cu_4I_4$ in the range of 20-300 K.

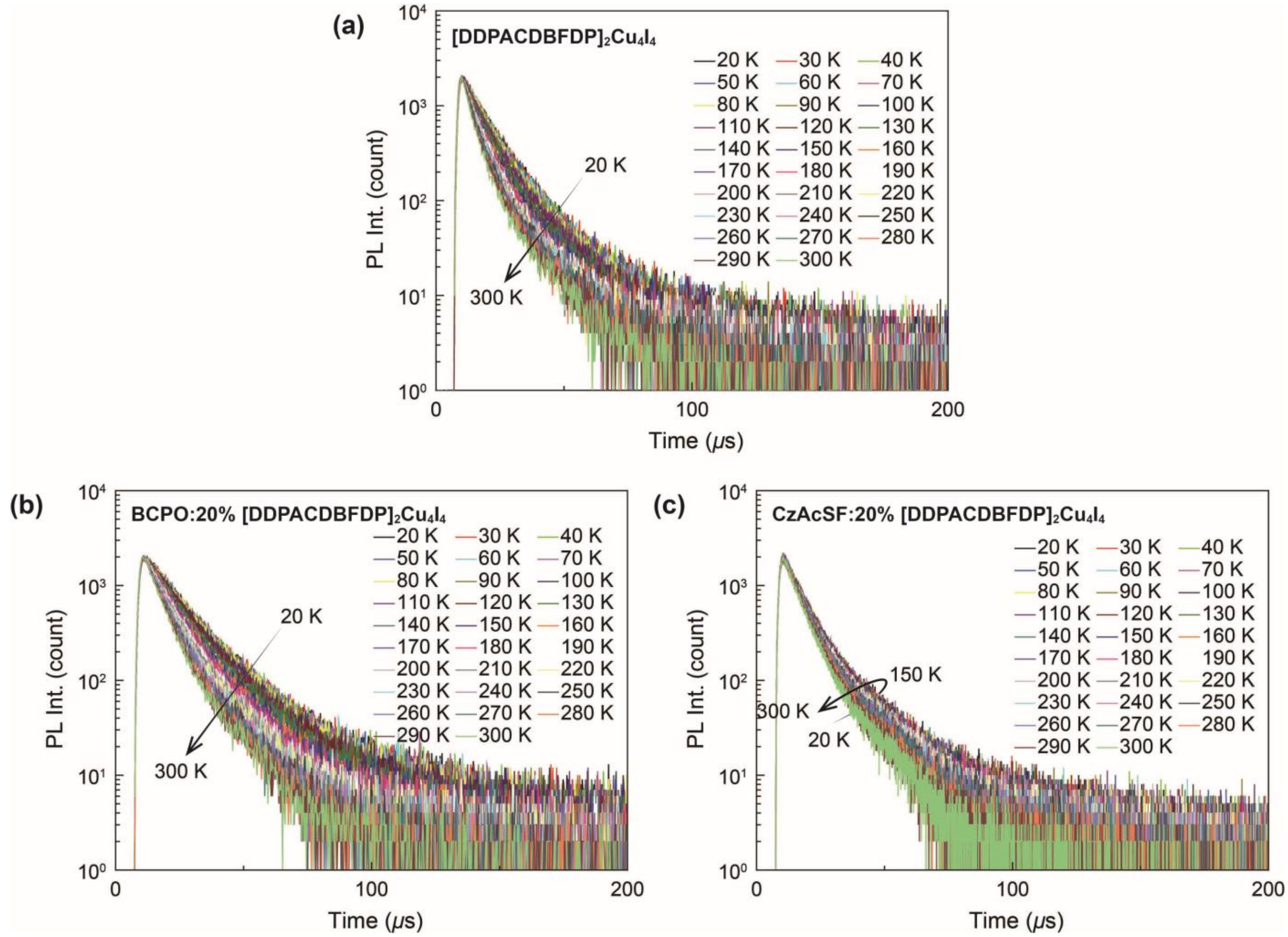

Supplementary Fig. 10 | Temperature dependence of time decays for neat (a), BCPO (b) and CzAcSF (c) hosted $[DDPACDBFDP]_2Cu_4I_4$ films in the range of 20-300 K. It is shown that at low temperature < 150 K, lifetimes of BCPO hosted films are the longest; while, neat films show the longer lifetimes than those of CzAcSF hosted films. At 20 K, CzAcSF:20% $[DDPACDBFDP]_2Cu_4I_4$ reveals a bi-exponential decay with τ_1 of

6.7 μs (66%) and τ_2 = 19.2 μs (34%); while, the neat cluster film has the longer lifetimes of τ_1 of 9.1 μs (88%) and τ_2 = 29.7 μs (12%). In comparison, the lifetimes of BCPO:20% $[DDPACDBFDP]_2Cu_4I_4$ at 20 K reach to τ_1 of 12.2 μs (88%) and τ_2 = 38.2 μs (12%). Different to neat and BCPO hosted films, even at 20 K, the shorter triplet lifetime of CzAcSF and energy transfer to the cluster render the shortest lifetimes of the cluster.

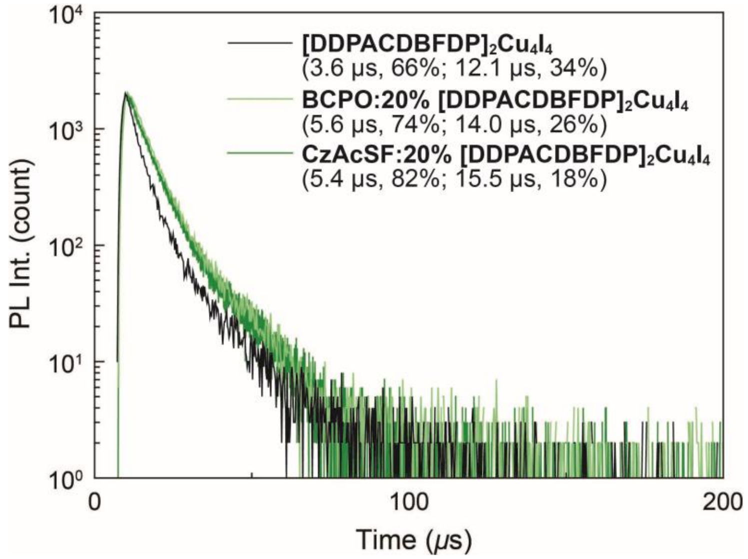

Supplementary Fig. 11 | Comparison on room-temperature time decays of neat, and BCPO and CzAcSF hosted $[DDPACDBFDP]_2Cu_4I_4$ films. The fitted lifetimes and corresponding proportions are listed in the legends. The incorporation of host matrixes induces the short lifetimes increased from 3.6 μs of neat film to ~5.5 μs, and long lifetimes increased from 12.1 μs of neat film to ~15.0 μs. Furthermore, using CzAcSF markedly increases the short-lifetime proportion, owing to the facilitated triplet-to-singlet conversion through external RISC effect by CzAcSF.

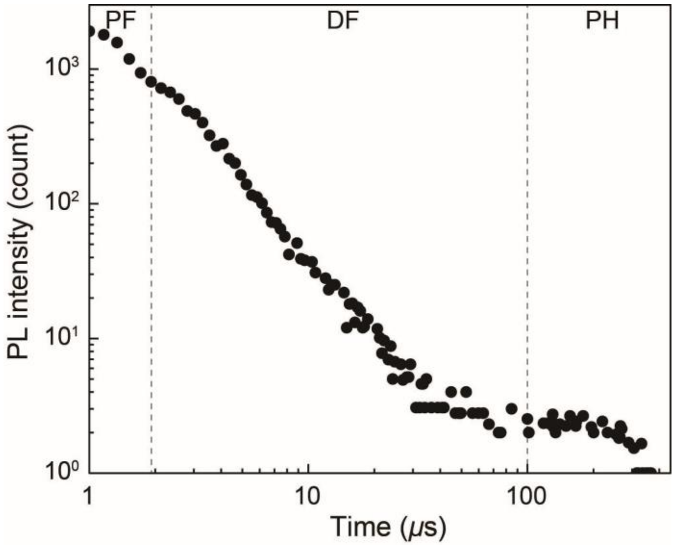

Supplementary Fig. 12 | High-resolution time decay of CzAcSF:20% $[DDPACDBFDP]_2Cu_4I_4$ film in the time range of 400 μs. Three decay stages can be recognized respectively at about 0-1, 1-100 and >100 μs, corresponding to prompt (PF) and delayed fluorescence (DF) and phosphorescence (PH).

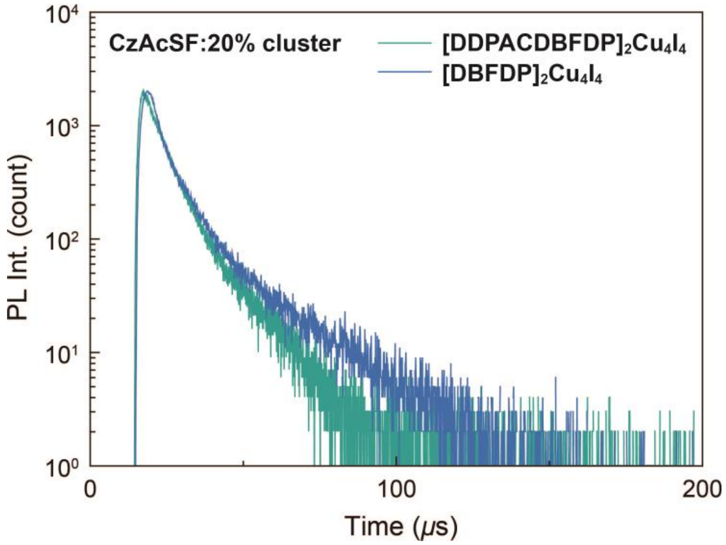

Supplementary Fig. 13 | Comparison on room-temperature time decays of CzAcSF:20% $[DBFDP]_2Cu_4I_4$ and $[DDPACDBFDP]_2Cu_4I_4$ films.

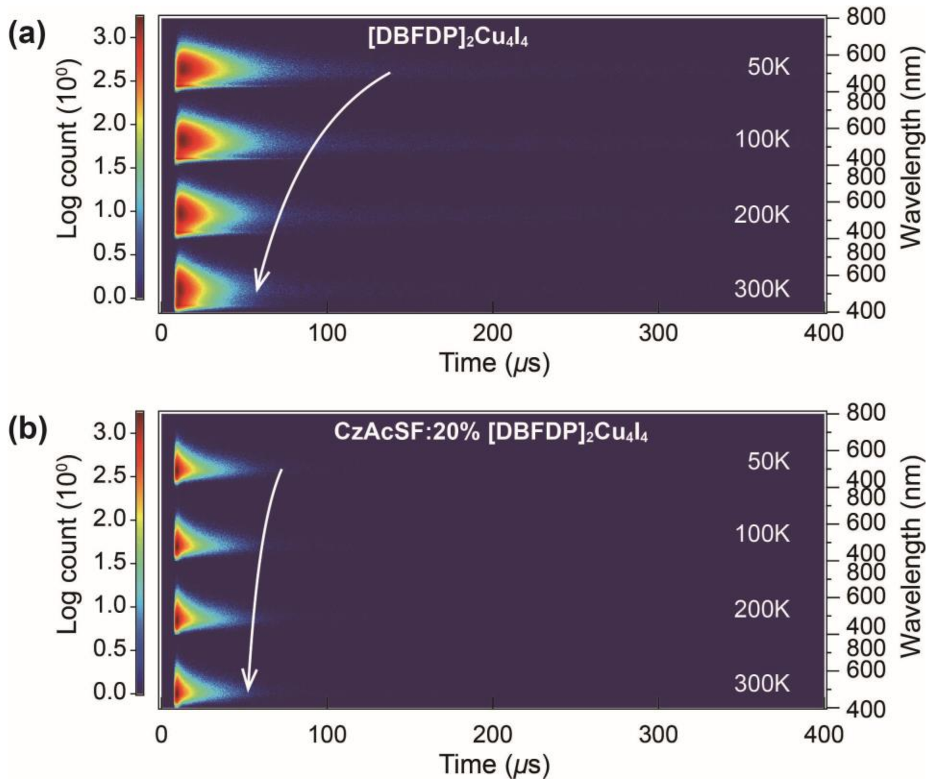

Supplementary Fig. 14 | Time resolved emission spectra (TRES) of neat film (a) and CzAcSF hosted films (b) based on $[DBFDP]_2Cu_4I_4$ in temperature range of 50-300 K and doping concentration range of 10-40%.

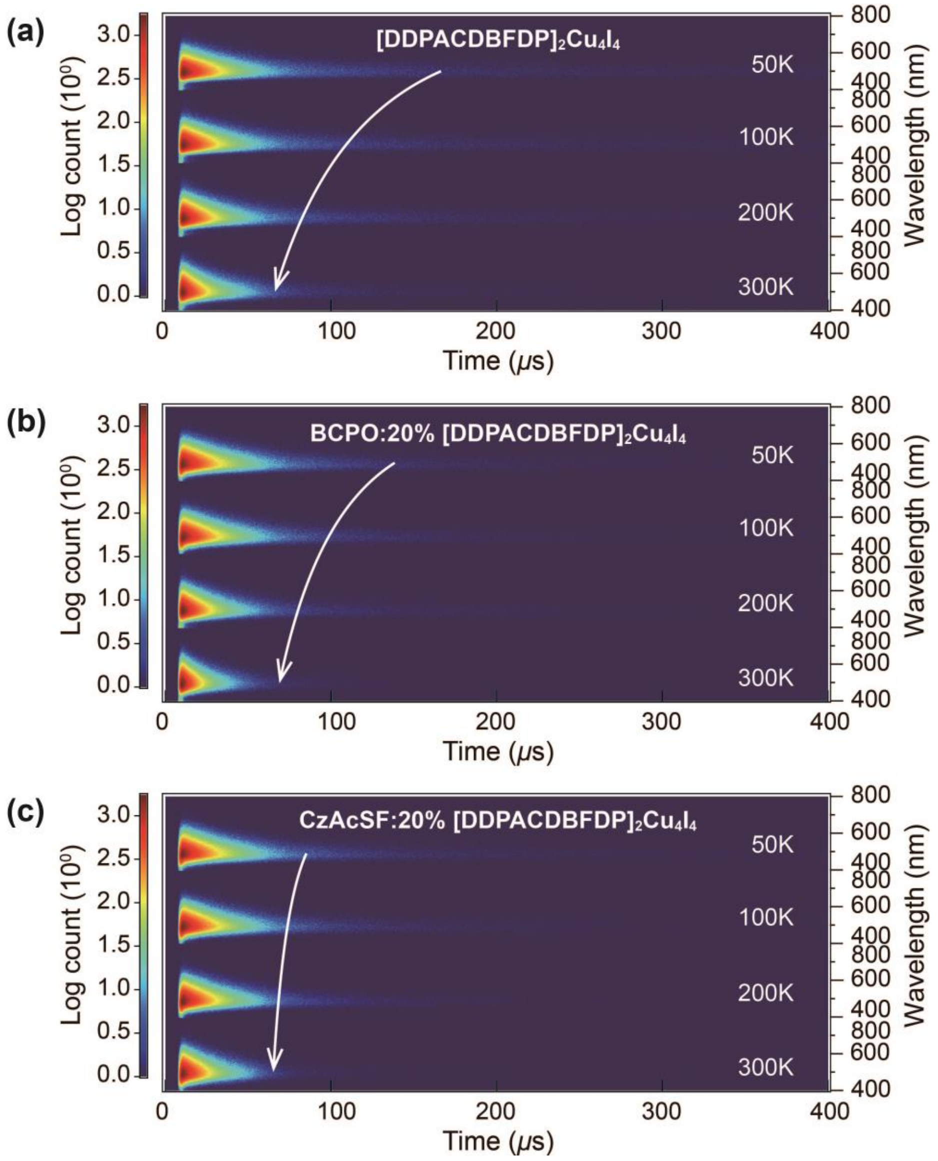

Supplementary Fig. 15 | Temperature-correlated time resolved emission spectra (TRES) of neat film (a), and BCPO (b) and CzAcSF hosted films (c) based on $[DDPACDBFDP]_2Cu_4I_4$ in the range of 50-300 K.

V. X-Ray luminescence Analysis

1. Room-temperature radioluminescence measurement

100 mg of the sample was pressed into 7 mm slices, and then gently placed into the sample tank to complete the sample loading. The voltage and current parameters to be tested are entered from the control panel and the radiation source was switched on. At the same time the spectrum scan was started. The spectrometer and radiation source were Edinburgh FS5 and Amptek Mini X-ray (4W), respectively.

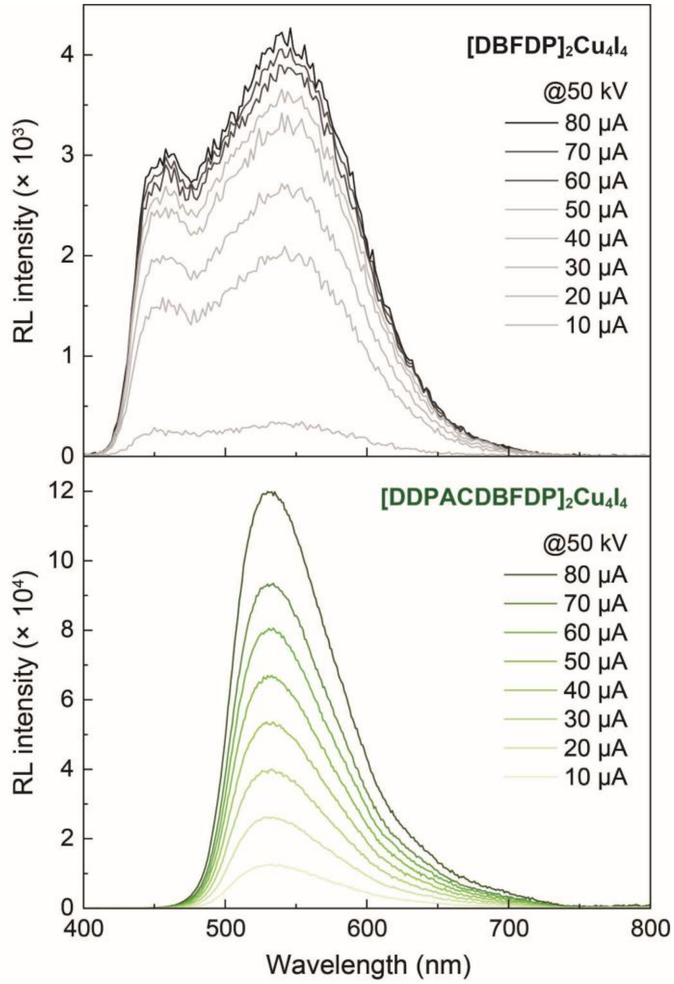

Supplementary Fig. 16 | Radioluminescence spectra of $[DBFDP]_2Cu_4I_4$ and $[DDPACDBFDP]_2Cu_4I_4$ at 50 kV under in the current range of 10-80 μA.

2. Temperature-dependent radioluminescence measurement

The 10 mg sample was spread gently onto the testing dish to cover the bottom. The test-bench temperature

was decreased to 80K and the radiation source parameters were set to 60 kV and 200 *μ*A via the control penal. Then, the radiation source was turned on, and spectral scanning was performed simultaneously. The scans were repeated following the operation above after ramping up to the target temperatures with a rate of 30 K/min. An Edinburgh spectrometer FLS980 and a radiation source of a MOXTEK MAGPRO 12W were used. While, the heat and cool table controller was a LINKAM HFS600.

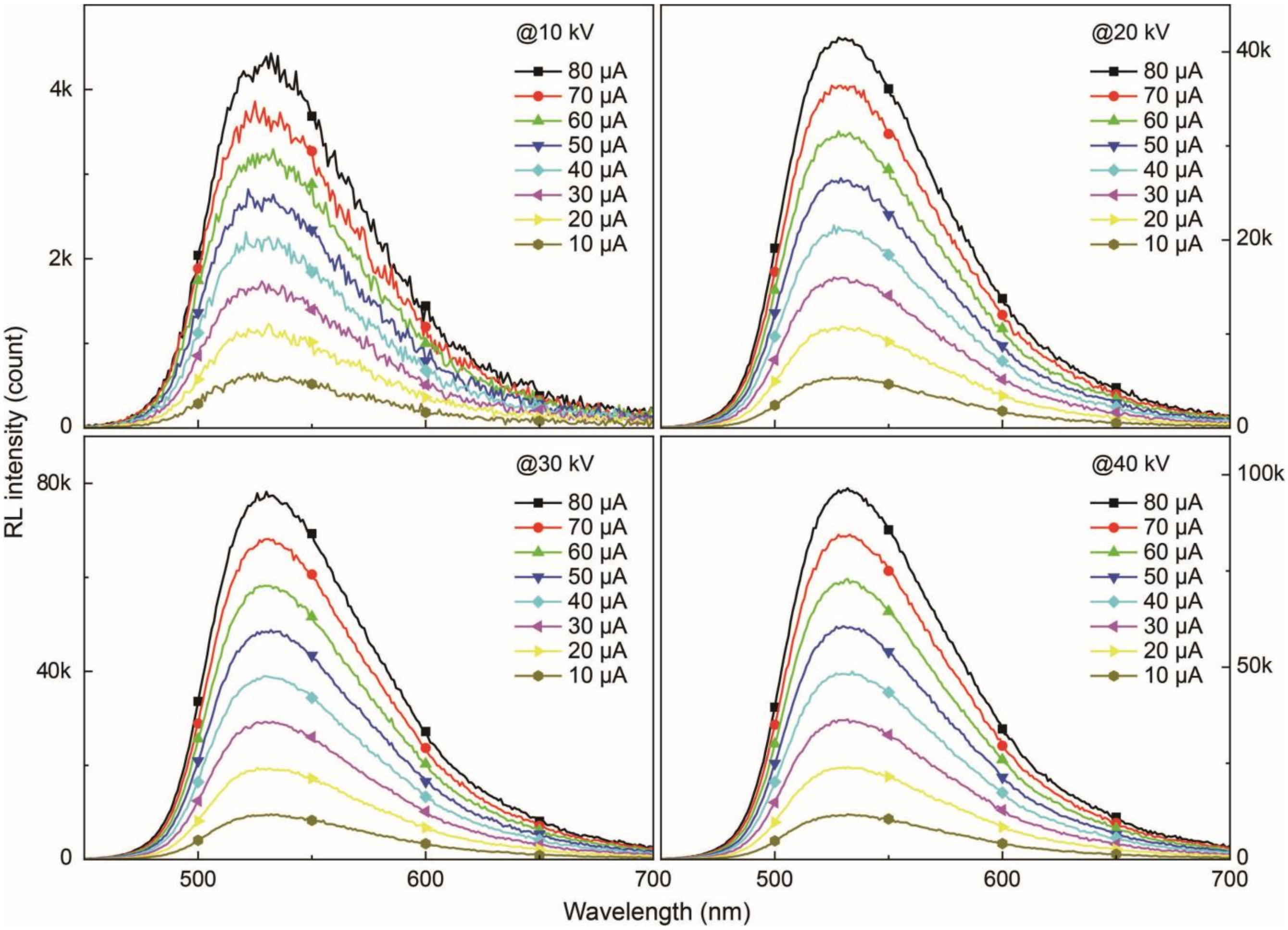

Supplementary Fig. 17 | Radioluminescence spectra of $[DDPACDBFDP]_2Cu_4I_4$ at 10, 20, 30 and 40 kV under in the current range of 10-80 μA.

3. X-ray imaging

The sample was mixed with PDMS at a mass ratio of 3:100 and then an appropriate amount of cyclohexane was added until the sample was completely dispersed. The PDMS initiator was then added at a mass ratio of 10:1. The mixture was treated into a uniform film. The radiation source, imaging object, scintillator film and camera were placed on the same level in turn.

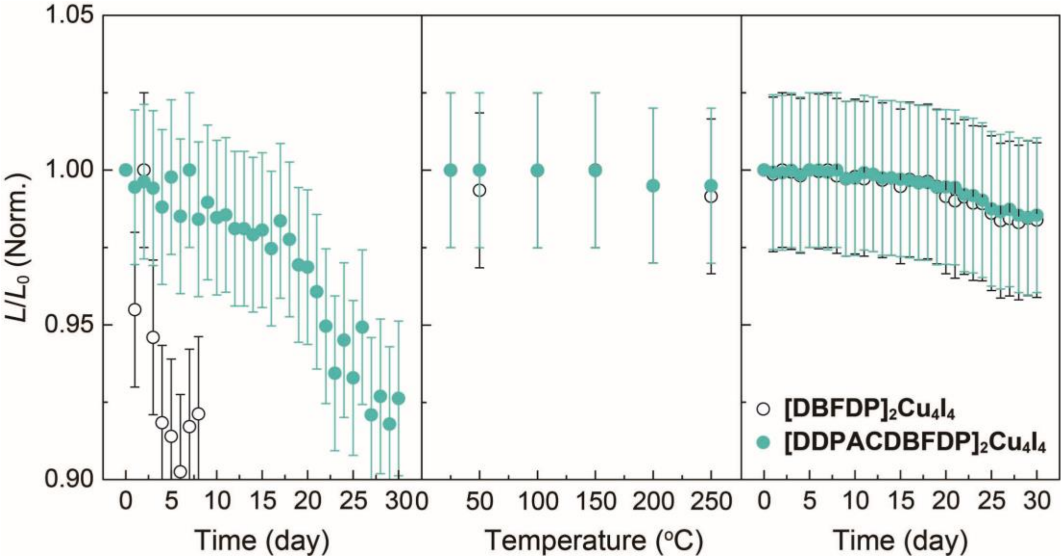

Supplementary Fig. 18 | Luminance variations of $[DDPACDBFDP]_2Cu_4I_4$ after exposed to UV light (365 nm) and embedded in water for one month and heated for ten minutes. After exposed to UV (365 nm) or water for one month or heated at 250 ^{o}C for ten minutes, luminance reduction of $[DDPACDBFDP]_2Cu_4I_4$ can be limited within 10% of initial intensities, indicating the outstanding stabilities under harsh conditions (Supplementary Fig. 15). The high photo- and thermo- stabilities of the cluster benefit the improvement of device duration for their CLEDs in electroluminescence process, since it is believed that device aging is mainly induced by photo- and thermo-degradation of the materials.

Table S1. Physical properties of $[DBFDP]_2Cu_4I_4$ and $[DDPACDBFDP]_2Cu_4I_4$.

cluster	$\lambda_{Abs.}$ (nm)	λ_{PL} (nm)	S_1[d] (eV)	T_1[e] (eV)	ΔE_{ST}[f] (eV)	f_S[g][h]	η_{PL}[i] (%)	T_g / T_m / T_d[j] (°C)	HOMO (eV)	LUMO (eV)
$[DDPACDBFDP]_2Cu_4I_4$	230, 287, 322[a]	510[b]	2.55	2.44	0.11	0.0156	71[b]	-/333/474	-5.57[k]	-3.22[k]
	252, 302, 391[b]	497[c]	2.69	2.66	0.03		95[c]		-5.06[h]	-1.89[h]
$[DBFDP]_2Cu_4I_4$	228, 291[a]	496[b]	3.13	2.97	0.14	0.0086	6[b]	209/-/315	-5.28[k]	-2.98[k]
	235, 290[b]	489[c]	2.83	2.79	0.04		52[c]		-5.07[h]	-1.56[h]

[a] In DCM solution (10^{-6} mol L^{-1}); [b] in film; [c] 20% doped CzAcSF films; [d] estimated according to onset wavelength of fluorescence peak; [e] estimated according to onset wavelength of phosphorescence peak; [f] singlet-triplet splitting; [g] singlet oscillator strength; [h] Gaussian simulation result; [i] photoluminescence quantum yield; [j] temperature at weight loss of 5%; [k] calculated according to cyclic voltammetric results.

Table S2. EL performance of OLEDs based on the clusters.

Cluster	Host	*x* (wt%)	*V*[a] (V)	L_{max}[b] (cd m^{-2})	*η*[c] η_{CE} (cd A^{-1})	η_{PE} (lm W^{-1})	η_{EQE} (%)	λ_{EL} (nm) / CIE (x, y)[d]
$[DDPACDBFDP]_2Cu_4I_4$	BCPO	10	6.0, 9.0, 12.7	1260	33.5, 29.1, 16.2	16.7, 10.1, 4.0	11.2, 9.8, 5.4	508/(0.24, 0.50)
		20	5.8, 8.6,12.1	1745	44.9, 39.2, 29.9	24.3, 14.3, 7.8	15.1, 13.3, 10.0	508/(0.24, 0.50)
		30	5.6, 7.6, 10.4	2002	39.3, 36.5, 27.1	22.0, 15.1, 8.2	13.2, 12.2, 9.1	508/(0.24, 0.50)
		40	5.4, 7.3, 9.5	2563	37.8, 34.4, 24.4	21.9, 14.8, 8.2	12.7, 11.6, 8.3	508/(0.24, 0.52)
$[DBFDP]_2Cu_4I_4$	BCPO	20	5.5, -, -	14	0.34, -, -	0.18, -, -	0.20, -, -	409, 448, 520 (0.27, 0.29)
$[DDPACDBFDP]_2Cu_4I_4$	CzAcSF	10	4.5, 6.4, 8.8	2058	31.2, 29.6, 20.8	21.4, 14.5, 7.7	11.1, 10.5, 7.5	500/(0.20, 0.44)
		20	3.6, 5.1, 7.0	3994	68.4, 67.3, 54.1	58.7, 41.7, 24.8	25.6, 25.1, 20.2	504/(0.21, 0.45)
		30	4.5, 6.3, 8.3	3264	46.0, 41.1, 31.5	32.1, 20.6, 12.1	16.4, 14.7, 11.3	504/(0.22, 0.50)
		40	4.3, 5.9,7.9	3719	33.6, 31.5, 23.5	24.5, 16.8, 9.4	12.0, 11.2, 8.4	508/(0.22, 0.50)
$[DBFDP]_2Cu_4I_4$	CzAcSF	20	3.8, 5.2, 7.8	1048	24.6, 18.7, 3.7	20.3, 12.2, 1.5	11.6, 8.8, 1.7	488/(0.16, 0.30)

[a] At 1, 100 and 1000 cd m^{-2}; [b] the maximum luminance; [c] EL efficiencies at the maximum, 100 and 1000 cd m^{-2}; [d] peak wavelengths and CIE coordinates of EL emissions at 1000 cd m^{-2}.

VI. Electroluminescence Analysis

1. Device fabrication

Before loading into a deposition chamber, the ITO substrate was cleaned with detergent and deionized water, dried in an oven at 120 °C for 4 h, and treated with oxygen plasma for 3 min. Poly(3,4-ethylenedioxythiophene): poly(styrenesulfonate) (PEDOT:PSS) layer (~40 nm) as hole transporting layer was spin-coated on the substrate. Then, cluster-doped emissive layers were spin-coated on PEDOT:PSS layer. The substrate was transferred to ultrahigh-vacuum chamber, and then electron transporting layers were evaporated at a rate of 0.1-0.2 nm s^{-1} sequentially at a pressure below 4×10^{-4} Pa. Onto the electron-transporting layer, a layer of LiF with 1-nm thickness was deposited at a rate of 0.1 nm s^{-1} to improve electron injection. Finally, a 100-nm layer of Al was deposited at a rate of 0.6 nm s^{-1} as the cathode. The emission area of the devices was 0.09 cm^2, as determined by the overlapped area of the anode and the cathode. After fabrication, devices were immediately transferred to a glove box for encapsulation with glass coverslips using epoxy glue.

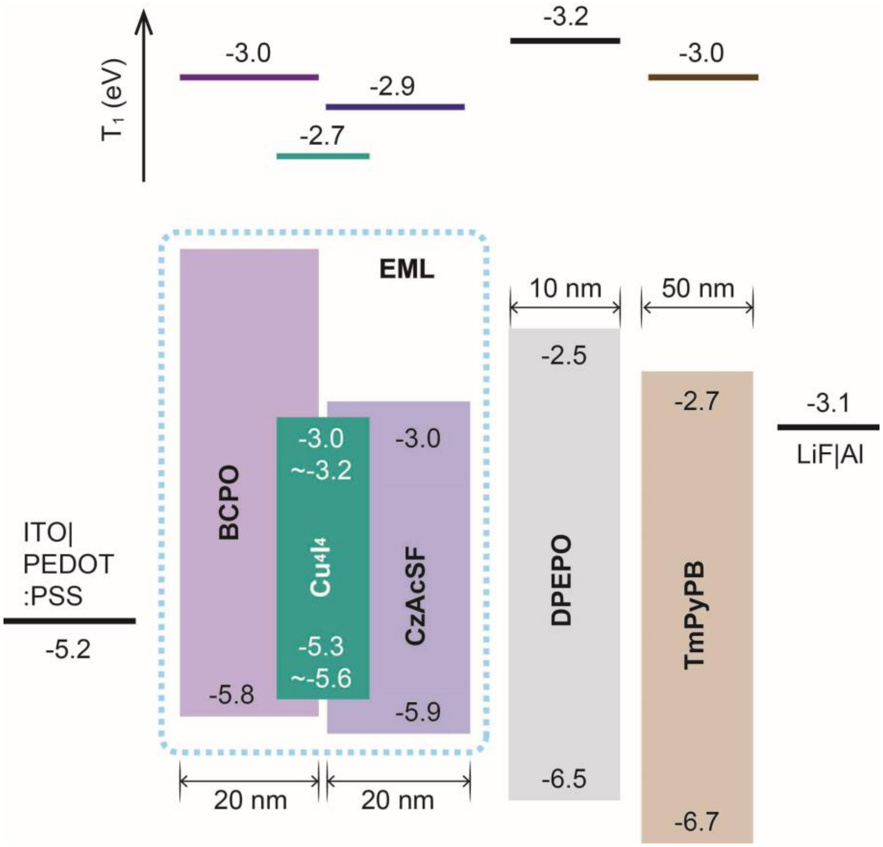

Supplementary Fig. 19 | Energy level diagram of CLEDs. Excitons were confined in emissive layers by engineering ionic potential, electron affinity and excited-state energy levels of each layer (Supplementary Fig. 18).

2. EL measurement

EL spectra and CIE coordinates were measured using a PR655 spectrum colorimeter. Current-density-voltage and brightness-voltage curves of the devices were measured using a Keithley 4200 source meter and a calibrated silicon photodiode. All measurements were carried out at room temperature under ambient conditions. For each structure, four devices were fabricated in parallel to confirm performance repeatability. The data reported herein were those closest to the average results. For transient EL measurement, the devices were loaded in an ultrahigh-vacuum chamber measured with Edinburgh FLS1000 equipped with a Tektronix AFG3022G function generator. The driving voltage was 5 V. The pulse width was 20 μs.

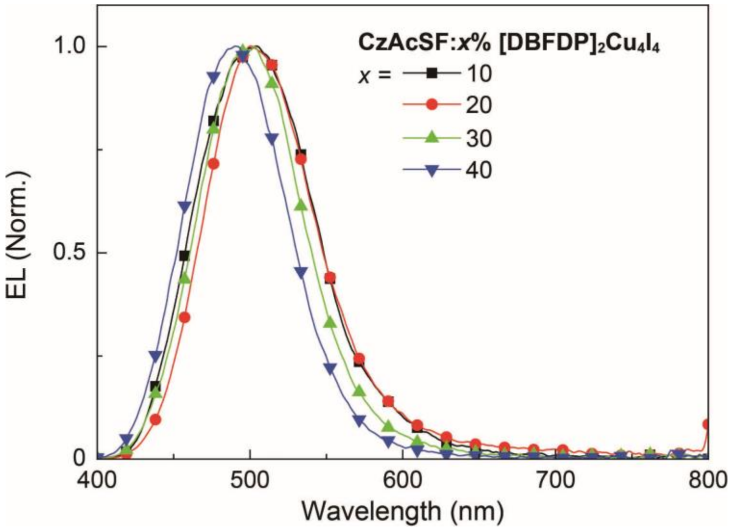

Supplementary Fig. 20 | Dependence of electroluminescence spectra for CzAcSF:$[DBFDP]_2Cu_4I_4$ based CLEDs on doping concentrations of 10-40%. BCPO:$[DBFDP]_2Cu_4I_4$ based devices showed white emission composed of blue and yellow bands, originated from M/ILCT and MICT, respectively (Fig. 4b and Table S2). In contrast, CzAcSF:$[DBFDP]_2Cu_4I_4$ showed single-band sky-blue electroluminescence emission (Supplementary Fig. 19). It means excitons were firstly formed on CzAcSF matrix, which then converted triplets to singlets, therefore limited triplet capture by MICT state.

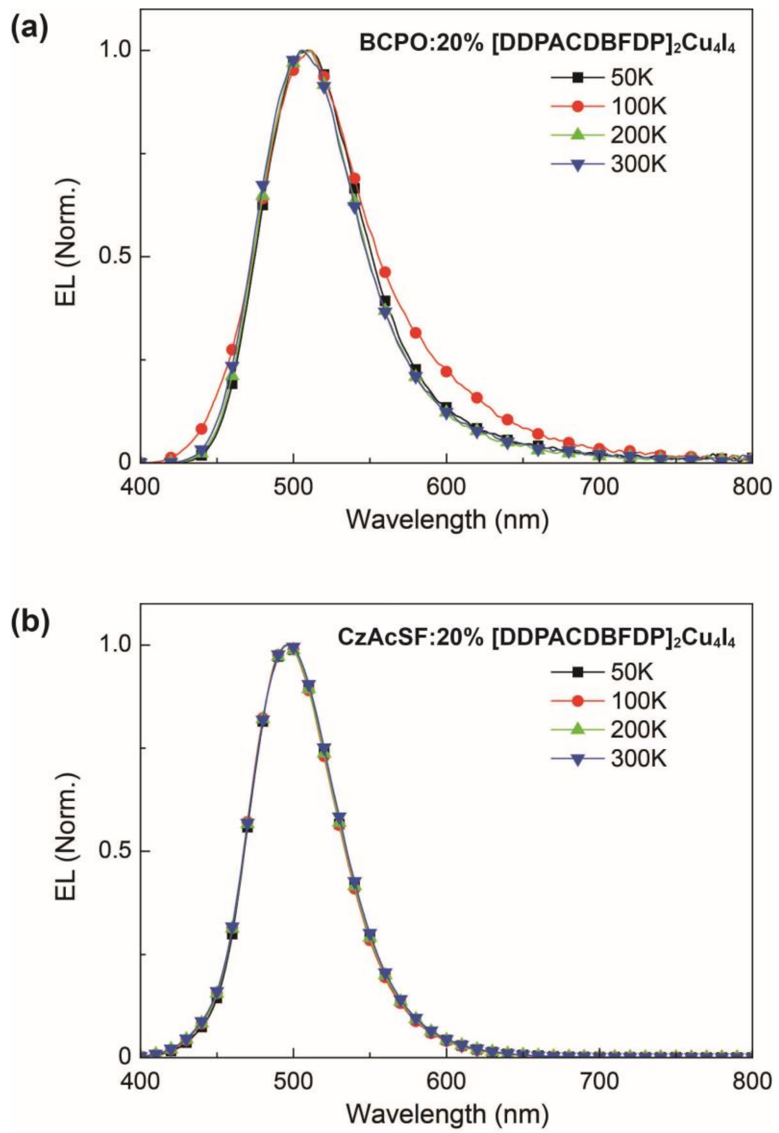

Supplementary Fig. 21 | Temperature dependence of electroluminescence spectra for BCPO:20% $[DDPACDBFDP]_2Cu_4I_4$ (a) and CzAcSF:20% $[DDPACDBFDP]_2Cu_4I_4$ (b) based CLEDs in the range of 50-300 K. Different to identical bluish green emissions of $[DDPACDBFDP]_2Cu_4I_4$ based CLEDs (Supplementary Fig. 20), using CzAcSF instead of BCPO made electroluminescence of $[DBFDP]_2Cu_4I_4$ changed from M/ILCT and MICT mixed white emission to M/ILCT predominant sky-blue emission (Fig. 4b, Supplementary Fig. 19 and Table S2).

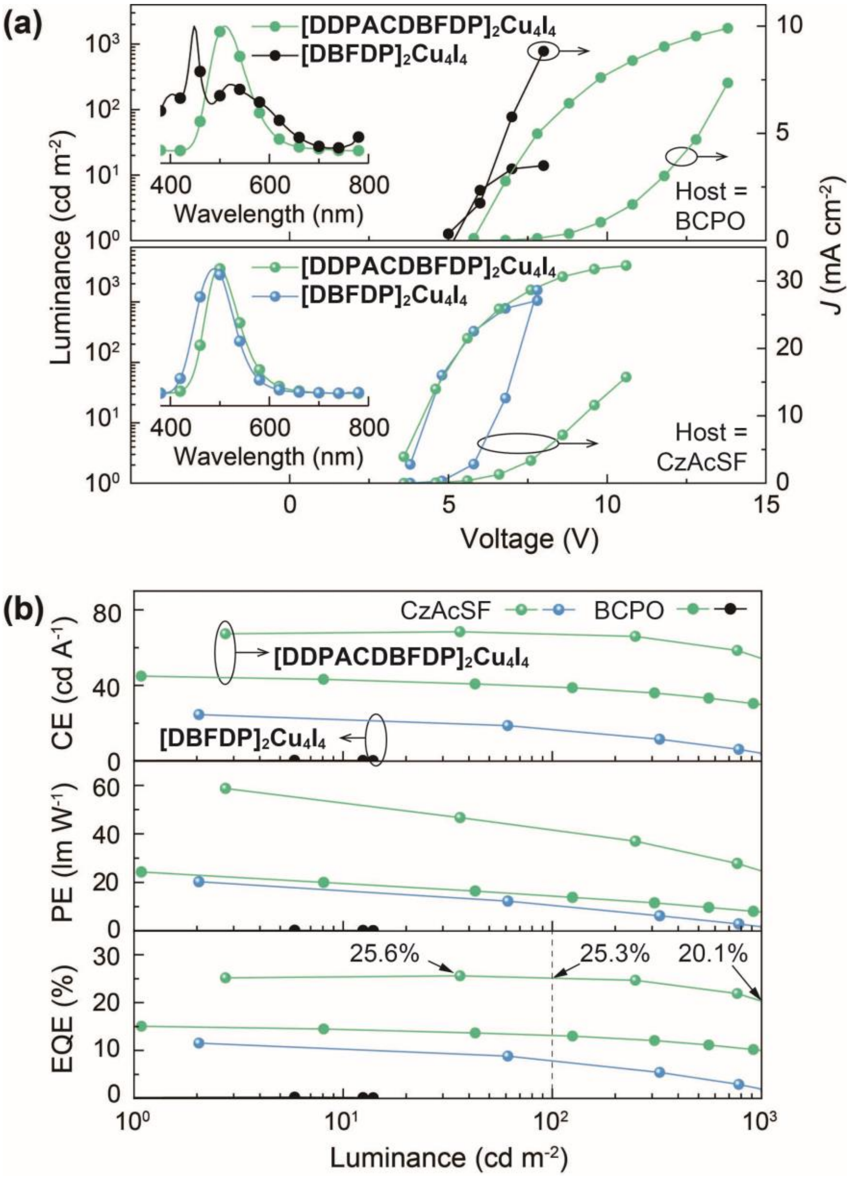

Supplementary Fig. 22 | (a) Luminance-current density (J)-voltage curves and electroluminescence spectra (insets) of CLEDs respectively using BCPO (top) and CzAcSF (bottom) as hosts. (b) Efficiencies *vs.* luminance relationships of the CLEDs. CE, PE and EQE refer to current efficiency, power efficiency and external quantum efficiency, respectively.

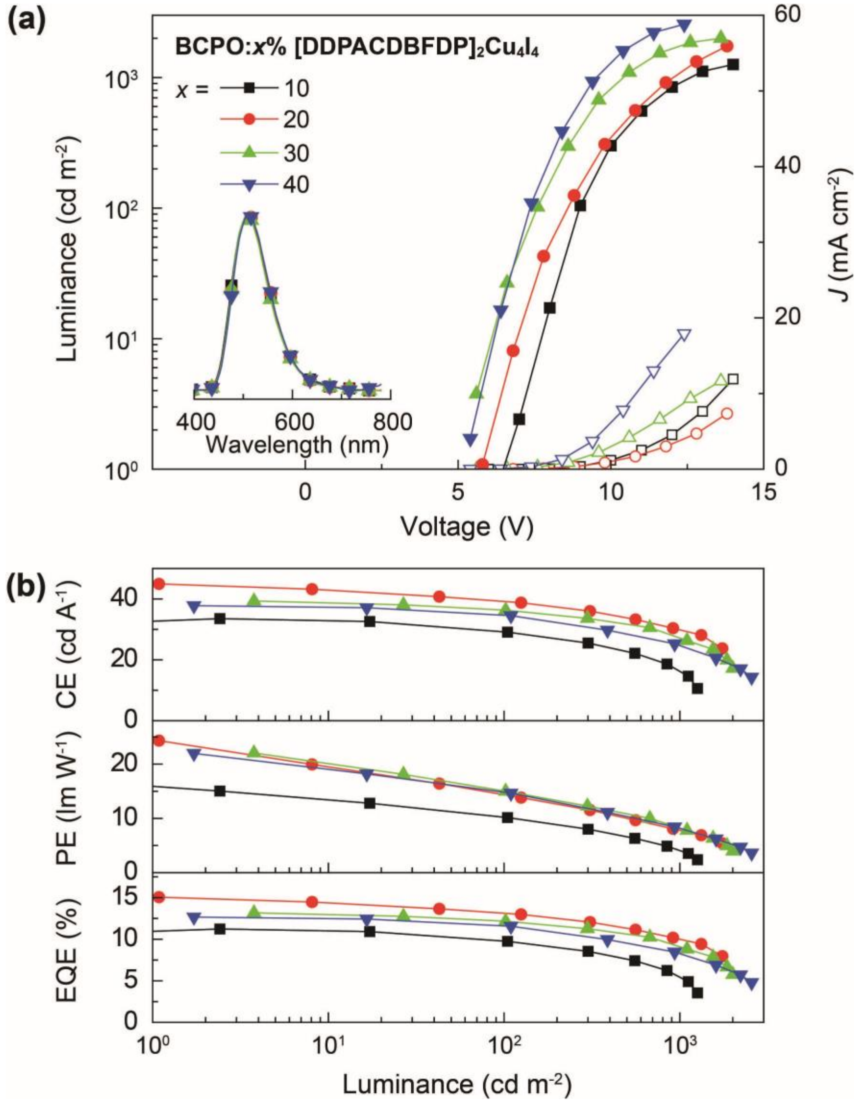

Supplementary Fig. 23 | (a) Brightness-current density (*J*)-voltage curves and electroluminescence spectra (b) luminance-efficiency curves of BCPO:*x*% [DDPACDBFDP]$_2$Cu$_4$I$_4$ based CLEDs.

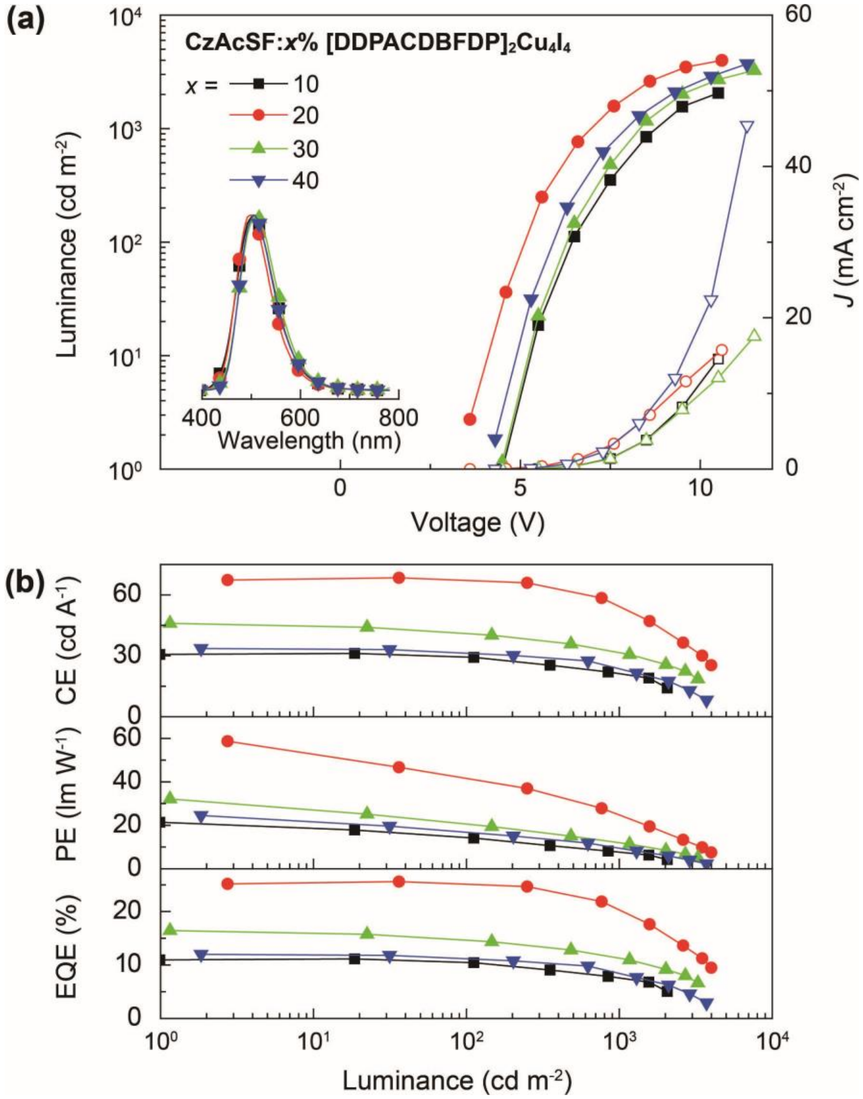

Supplementary Fig. 24 | (a) Brightness-current density (J)-voltage curves and electroluminescence spectra (b) luminance-efficiency curves of CzAcSF:x% $[DDPACDBFDP]_2Cu_4I_4$ based CLEDs. At 1000 nits, CzAcSF:$[DDPACDBFDP]_2Cu_4I_4$ still achieved η_{EQE} over 20%, corresponding to a record-low EQE roll-off of 23% among CLEDs.

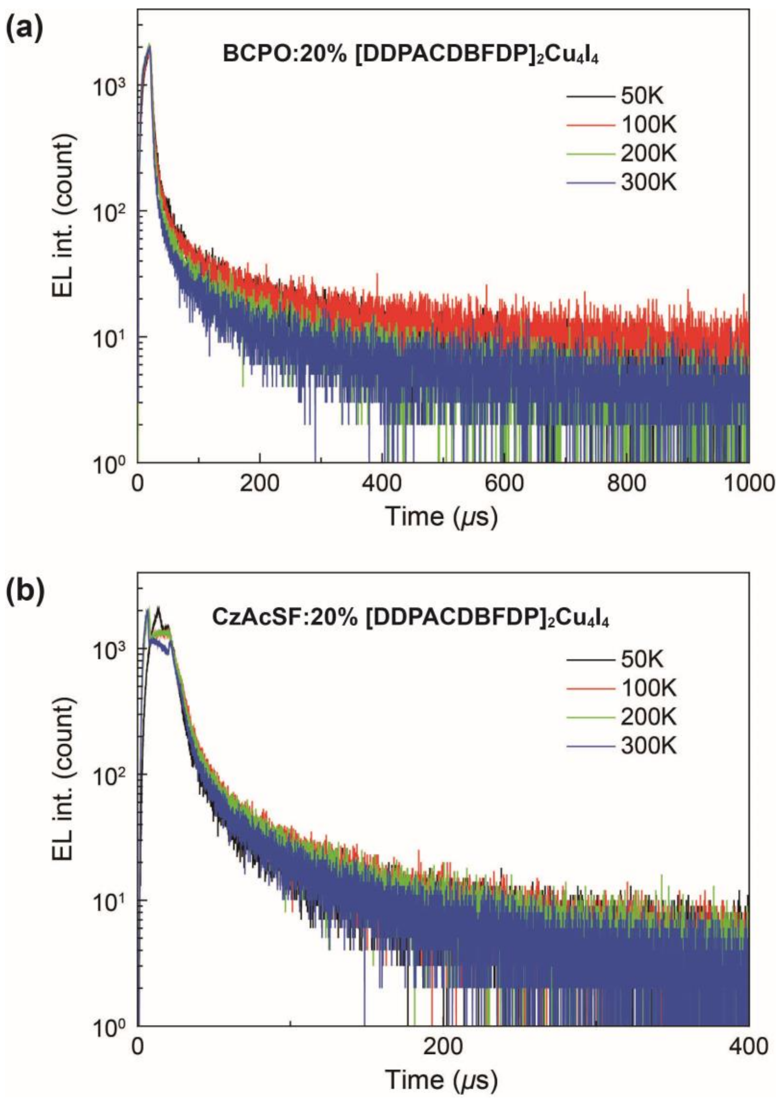

Supplementary Fig. 25 | Temperature-dependent time decays of (a) BCPO:20%$[DDPACDBFDP]_2Cu_4I_4$ and (b) CzAcSF:20% $[DDPACDBFDP]_2Cu_4I_4$ based CLEDs. Electroluminescence decays of the devices are bi-exponential. Electroluminescence lifetimes of BCPO:20% $[DDPACDBFDP]_2Cu_4I_4$ are 6.8 (45%) and 173.5 (55%) μs at 50 K, 6.6 (47%) and 151.3 (53%) μs at 100 K, 4.5 (57%) and 128.6 (43%) μs at 200 K, 3.7 (59%) and 97.1 (41%) μs at 300 K, respectively. Electroluminescence lifetimes of CzAcSF:20% $[DDPACDBFDP]_2Cu_4I_4$ are 3.0 (71%) and 26.3 (29%) μs at 50 K, 7.8 (65%) and 59.2 (35%) μs at 100 K, 7.0 (61%) and 55.5 (39%) μs at 200 K, 6.5 (60%) and 52.0 (40%) μs at 300 K, respectively. It is shown that along with temperature increasing, both short and long lifetimes of BCPO hosted devices are reduced. In contrast, electroluminescence lifetime of CzAcSF hosted devices increased from 50 to 100 K, and then decreased at > 100K.

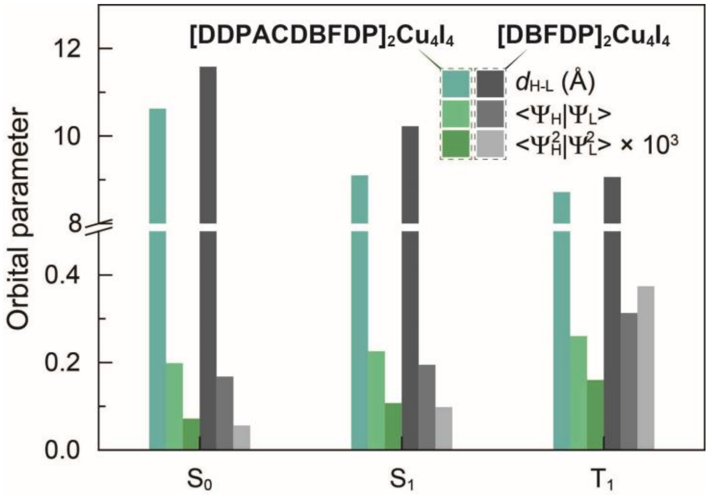

Supplementary Fig. 26 | Comparison on centroid distance ($d_{H\text{-}L}$), wave function overlap integral ($<\Psi_H|\Psi_L>$) and electronic cloud overlap probabilities ($<\Psi_H^2|\Psi_L^2>$) of the frontier orbitals of S_0, the S_1 and T_1 states of the clusters. The parameters of the clusters at the same states are put together for clarity.

VII. References

1 Xie, M., Han, C., Zhang, J., Xie, G. & Xu, H. White Electroluminescent Phosphine-Chelated Copper Iodide Nanoclusters. *Chem. Mater.* **29**, 6606-6610, doi:10.1021/acs.chemmater.7b01443 (2017).

2 Becke, A. D. Density-functional thermochemistry. III. The role of exact exchange. *J. Chem. Phys.* **98**, 5648-5652 (1993).

3 Lee, C., Yang, W. & Parr, R. G. Development of the Colle-Salvetti correlation-energy formula into a functional of the electron density. *Phys. Rev. B* **37**, 785-789 (1988).

4 Martin, R. L. Natural transition orbitals. *J. Chem. Phys.* **118**, 4775-4777, doi:10.1063/1.1558471 (2003).

5 Gaussian 09 v. D. 1 (Gaussian, Inc., Wallingford CT, USA, 2009).
